# Supplementary material for: The AAA+ chaperone VCP disaggregates Tau fibrils and generates aggregate seeds in a cellular system
Source: Nat Commun. 2023 Feb 2;14:560. doi: 10.1038/s41467-023-36058-2 (PMC9894937; doi:10.1038/s41467-023-36058-2)
Supplement: Supplementary file 1 — Supplementary Information [file 41467_2023_36058_MOESM1_ESM.pdf]

Supplementary Information

The AAA+ chaperone VCP disaggregates Tau fibrils and generates aggregate seeds in a cellular system

Itika Saha[1,2], Patricia Yuste-Checa[1,2], Miguel Da Silva Padilha[3,4,7], Qiang Guo[5,#], Roman Körner[1], Hauke Holthusen[1], Victoria A. Trinkaus[1,5,6], Irina Dudanova[3,4,7], Rubén Fernández-Busnadiego[2,5,8,9], Wolfgang Baumeister[5], David W. Sanders[10,‡], Saurabh Gautam[1,§], Marc I. Diamond[10], F. Ulrich Hartl[1,2,6,*] and Mark S. Hipp[1,6,11,12*]

[1]Department of Cellular Biochemistry, Max Planck Institute of Biochemistry, Am Klopferspitz 18, 82152 Martinsried, Germany.

[2]Aligning Science Across Parkinson's (ASAP) Collaborative Research Network, Chevy Chase, MD, USA.

[3]Molecular Neurodegeneration Group, Max Planck Institute for Biological Intelligence, 82152 Martinsried, Germany.

[4]Department of Molecules – Signaling – Development, Max Planck Institute for Biological Intelligence, Am Klopferspitz 18, 82152 Martinsried, Germany.

[5]Department of Structural Molecular Biology, Max Planck Institute of Biochemistry, Am Klopferspitz 18, 82152 Martinsried, Germany.

[6]Munich Cluster for Systems Neurology (SyNergy), Munich, Germany.

[7]Center for Anatomy, Faculty of Medicine and University Hospital Cologne, University of Cologne, 50931 Cologne, Germany.

[8]Institute of Neuropathology, University Medical Center Göttingen, 37099 Göttingen, Germany.

[9]Cluster of Excellence "Multiscale Bioimaging: from Molecular Machines to Networks of Excitable Cells" (MBExC), University of Göttingen, Germany.

[10]Center for Alzheimer's and Neurodegenerative Diseases, Peter O'Donnell Jr. Brain Institute, University of Texas Southwestern Medical Center, Dallas, 75390 Texas, USA.

[11]School of Medicine and Health Sciences, Carl von Ossietzky University Oldenburg, Oldenburg, Germany.

[12]Department of Biomedical Sciences of Cells and Systems, University Medical Center Groningen, University of Groningen, Antonius Deusinglaan, 1, 9713 AV Groningen, The Netherlands.

# Present address: State Key Laboratory of Protein and Plant Gene Research, School of Life Sciences and Peking-Tsinghua Center for Life Sciences, Peking University, Beijing 100871, China.

‡ Present address: Department of Chemical and Biological Engineering, Princeton University, Princeton, NJ 08544, USA

§ Present address: Boehringer Ingelheim International GmbH, 55216 Ingelheim, Germany and ViraTherapeutics GmbH, 6063 Rum, Austria.

*Correspondence to uhartl@biochem.mpg.de and m.s.hipp@umcg.nl

Supplementary information includes 13 Supplementary Figures, Supplementary Table 1 and uncropped scans of blots presented in supplementary figures.

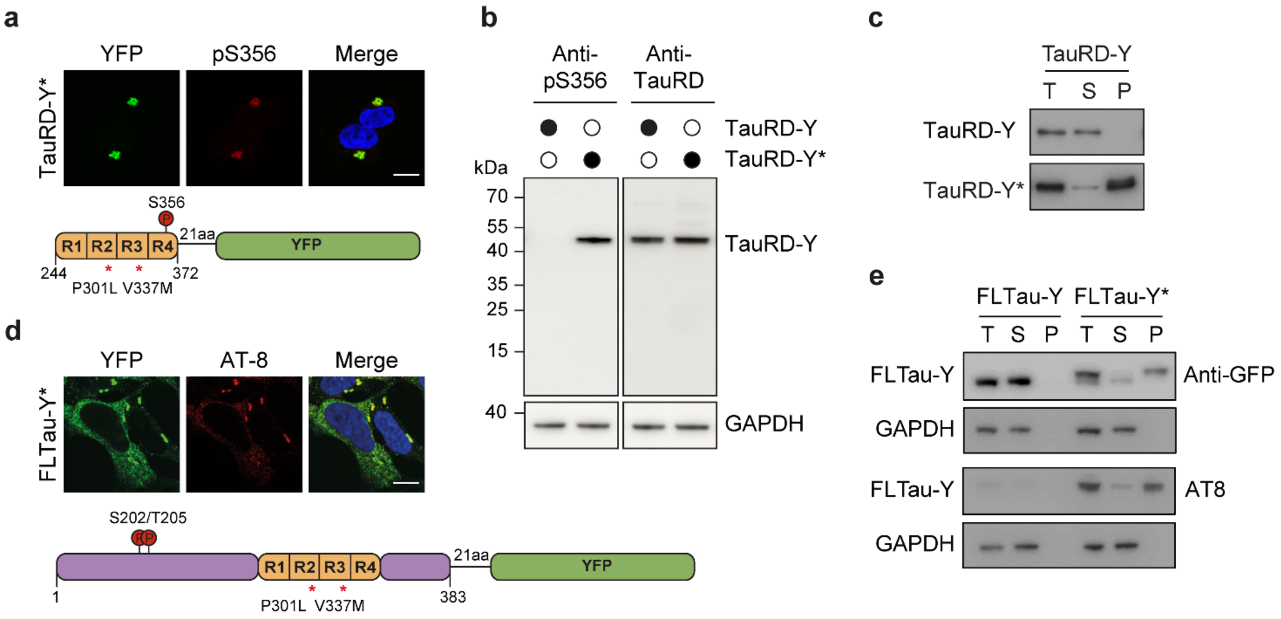

Supplementary Fig. 1: Tau aggregation in a constitutive expression model.

a Immunofluorescence staining of TauRD-Y* cells with an antibody against Tau phosphorylation at S356 (red) and YFP fluorescence of TauRD-Y (green). Scale bar, 10 µm. **b** Analysis of Tau S356 phosphorylation in lysates of TauRD-Y and TauRD-Y* cells by immunoblotting. Total TauRD-Y was detected using antibody against TauRD. **c** Solubility of TauRD-Y in TauRD-Y and TauRD-Y* cells at steady state, determined by fractionation of cell lysate by centrifugation, followed by immunoblotting with anti-GFP antibody. T, total cell lysate, S, supernatant, P, pellet. **d** Immunofluorescence staining of full-length Tau (FLTau-Y) in aggregate-containing FLTau-Y* cells with AT-8 antibody specific for Tau phosphorylation at S202/T205 (red) and YFP fluorescence of TauRD-Y (green). Scale bar, 10 µm. **e** Solubility of phosphorylated FLTau-Y in FLTau-Y and FLTau-Y* cells at steady state analyzed as in (c). Immunoblotting was with AT-8 antibody (bottom) and anti-GFP (top). GAPDH served as loading control.

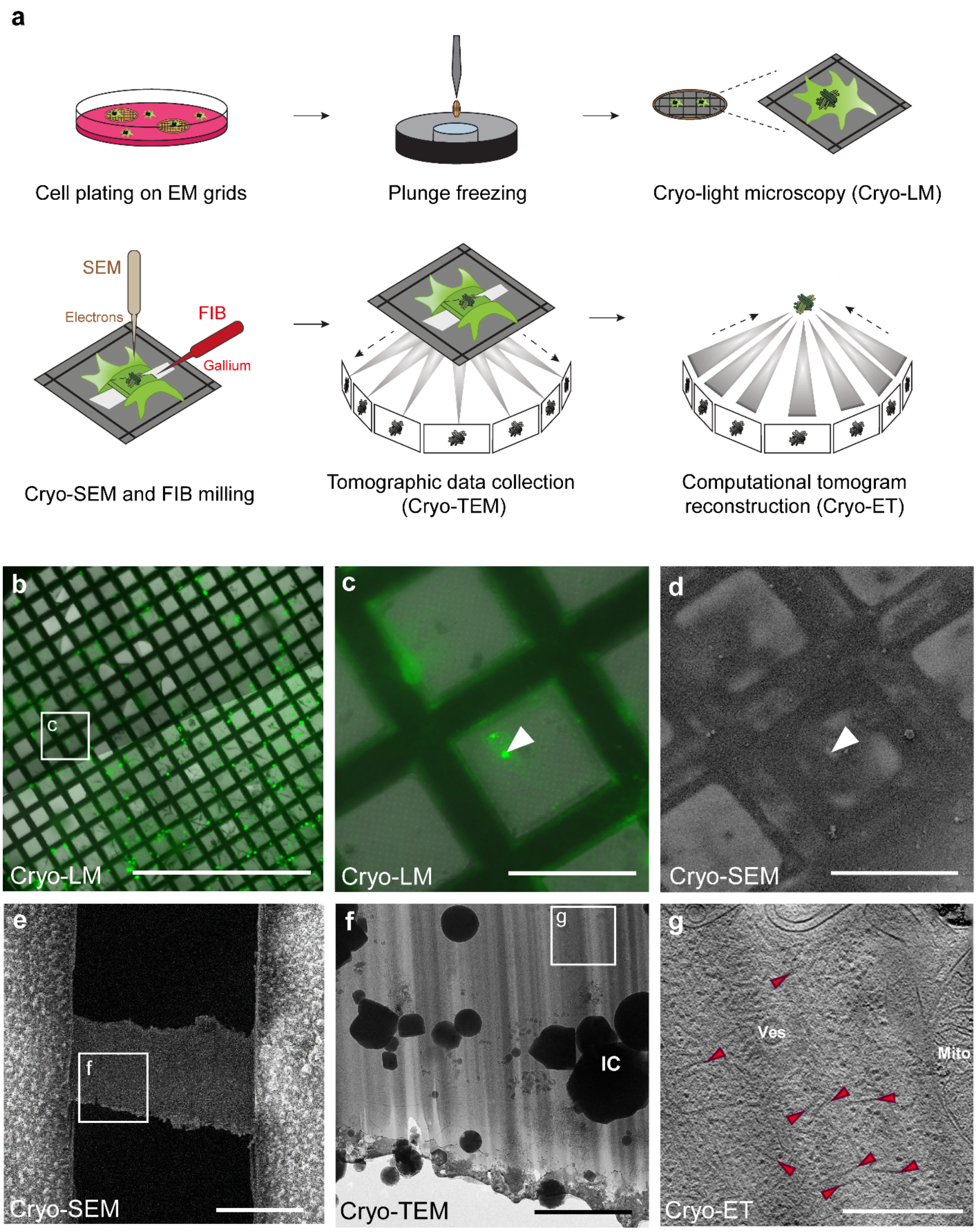

Supplementary Fig. 2: Cryo-correlative-light-electron microscopy (cryo-CLEM) workflow.

a Schematic representation of the cryo-CLEM workflow on cells containing TauRD-Y aggregates. Cryo-LM (cryo-light microscopy), cryo-SEM (cryo-scanning electron microscopy), cryo-FIB

(focused ion beam), cryo-TEM (cryo-transmission electron microscopy), cryo-ET (cryo-electron tomography). **b** Mouse primary neurons were cultured on EM grids for 10 days and transduced with TauRD-Y followed by treatment with aggregate-containing cell lysates. 6 days after transduction, grids were vitrified by plunge freezing. Thereafter, grids were imaged by cryo-light microscopy (cryo-LM). An image of a grid with neurons containing aggregates is shown. Scale bar, 1 mm. **c** Zoomed in cryo-LM image of the area marked in (b) by a white box. The arrowhead indicates a neuronal TauRD-Y inclusion. Scale bar, 100 µm. **d** EM grids were transferred into the cryo-FIB/SEM. Cryo-SEM image of the area shown in (c). The arrowhead indicates the same location of the area shown in (c). Scale bar, 100 µm. **e** A ~200 nm thick lamella was generated at that location by cryo-FIB milling. Scale bar, 10 µm. **f** Cryo-TEM overview of the lamella region marked in (e). IC: Ice crystal. Scale bar, 2 µm. **g** 1.4 nm thick tomographic slice of a neuronal TauRD-Y inclusion recorded in the area marked in (f). Red arrowheads indicate representative TauRD-Y fibrils. Ves: Vesicle, Mito: Mitochondrion. Scale bar, 500 nm.

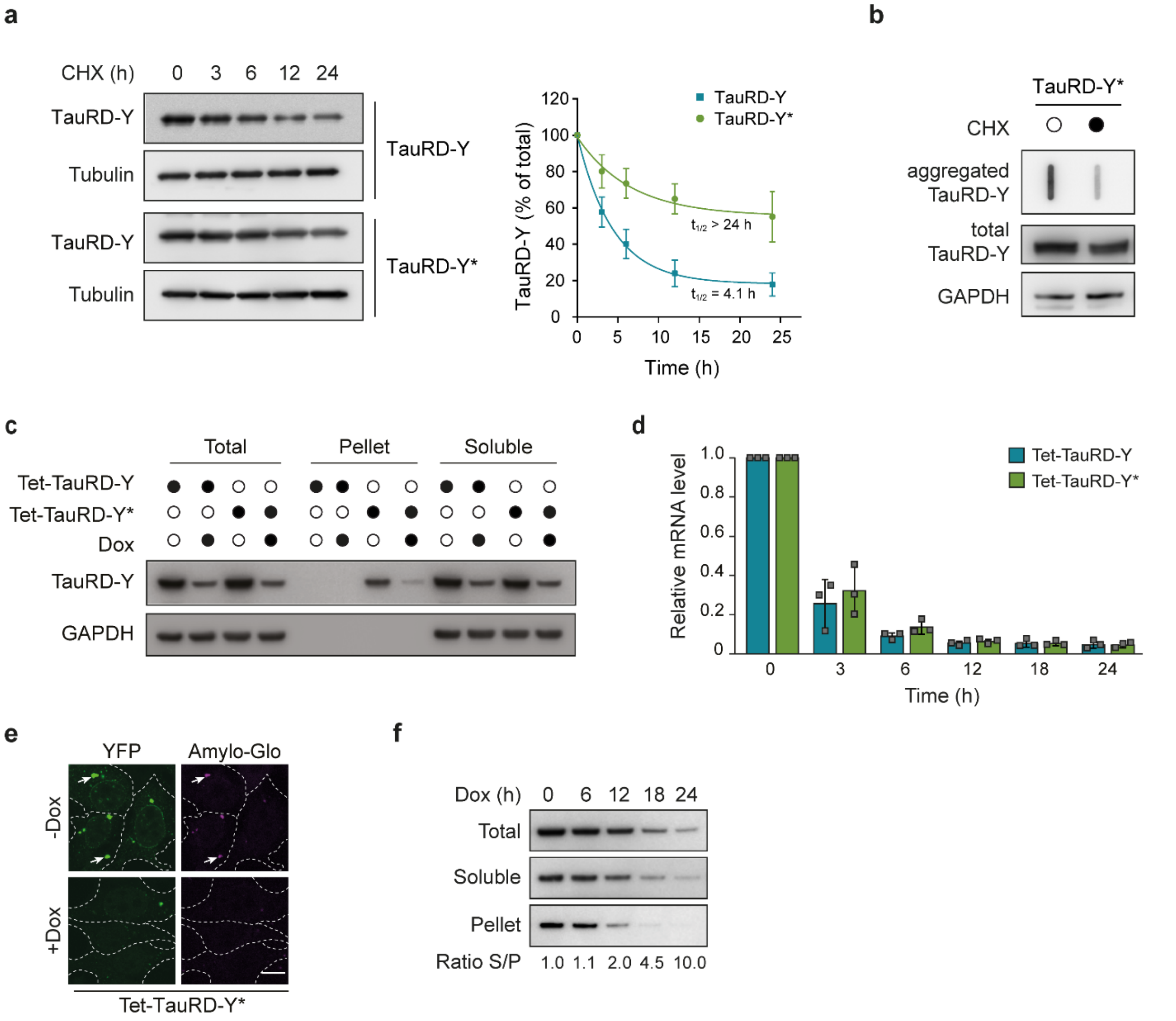

Supplementary Fig. 3: TauRD-Y aggregation and clearance upon inhibition of expression in a constitutive and Tet-regulated TauRD-Y expression system.

a Turnover of TauRD-Y in TauRD-Y and TauRD-Y* cells upon cycloheximide (CHX) shut-off (CHX; 50 µg/mL). Left, anti-GFP immunoblots to determine TauRD-Y levels. Tubulin served as loading control. Right, exponential fits of CHX chase data and corresponding half-lives ($t_{1/2}$). Mean ± s.d.; n=3. **b** Filter trap analysis of aggregated TauRD-Y upon CHX chase for 24 h. Aggregated and total TauRD-Y levels were determined by anti-GFP immunoblotting. GAPDH served as loading control. **c** Solubility of TauRD-Y in Tet-TauRD-Y and Tet-TauRD-Y* cells upon addition of 50 ng/mL doxycycline (Dox) for 24 h. Cell lysates were fractionated as in

Supplementary Fig. 1c. TauRD-Y was detected with anti-GFP antibody. GAPDH served as loading control. **d** Quantitative PCR analysis of TauRD-Y mRNA in Tet-TauRD-Y and Tet-TauRD-Y* cells treated with Dox for 0, 3, 6, 12, 18 and 24 h. mRNA levels were normalized to the reference gene *RPS18*. Mean ± s.d.; n=3. **e** Representative fluorescence images of Tet-TauRD-Y* cells treated with Dox for 24 h showing staining of TauRD-Y inclusions (green) with Amylo-Glo (magenta). White dashed lines indicate cell boundaries. Scale bar, 10 µm. **f** Solubility of TauRD-Y in Tet-TauRD-Y* cells upon addition of Dox for the indicated times. Normalized ratios of TauRD-Y in soluble (S) and pellet (P) fractions are stated. Source data are provided as a Source Data file.

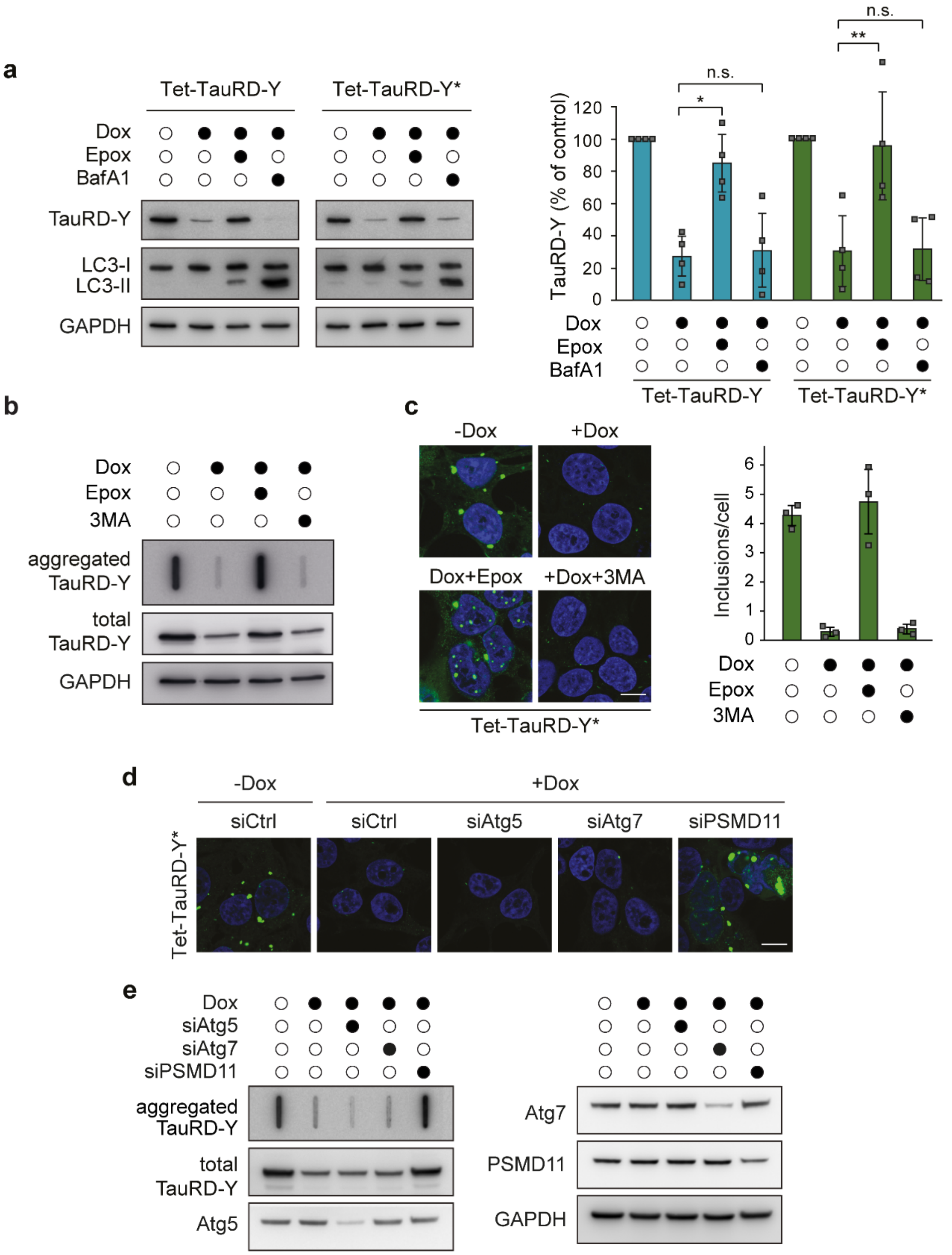

Supplementary Fig. 4: Effect of UPS and autophagy inhibition on TauRD-Y levels and aggregate clearance.

a Analysis of TauRD-Y levels in Tet-TauRD-Y and Tet-TauRD-Y* cells treated for 24 h with doxycycline (Dox; 50 ng/mL) alone or in combination with Epoxomicin (Epox; 50 nM) or

Bafilomycin A1 (BafA1; 50 nM). TauRD-Y and LC3 levels were determined by immunoblotting against GFP and LC3B respectively. GAPDH served as loading control. Mean ± s.d.; n=4. *p<0.05 (Tet-TauRD-Y: + Dox vs + Dox + Epox, p=0.0114), **p<0.01 (Tet-TauRD-Y*: + Dox vs + Dox + Epox, p=0.0026); n.s. non-significant (Tet-TauRD-Y: + Dox vs + Dox + Epox, p= 0.6422; Tet-TauRD-Y*: + Dox vs + Dox + Epox, p= 0.8799) from two-tailed Student's paired t-test. **b** Filter trap analysis of Tet-TauRD-Y* cells treated for 24 h with Dox alone or in combination with Epoxomicin (Epox; 50 nM) or 3-methyladenine (3MA; 5 mM). Aggregated and total TauRD-Y was detected with anti-GFP antibody. **c** Left, representative images of Tet-TauRD-Y* cells treated for 24 h with Dox alone or, in combination with Epoxomicin (Epox; 50 nM) or 3MA (5 mM). Scale bar, 10 μm. Right, quantification of TauRD-Y foci. 100-200 cells analyzed per experiment. Mean ± s.d.; n=3. **d** Representative images of Tet-TauRD-Y* cells transfected with non-targeted (Ctrl) siRNA or siRNA against Atg5 (50 nM), Atg7 (50 nM) and PSMD11 (25 nM). 72 h after transfection, doxycycline (Dox; 50 ng/mL) was added for another 24 h where indicated. Scale bar, 10 μm. **e** Filter trap analysis of Tet-TauRD-Y* cells transfected with siRNAs and treated with Dox as stated in (d). TauRD-Y was detected by immunoblotting with anti-GFP antibody. Source data are provided as a Source Data file.

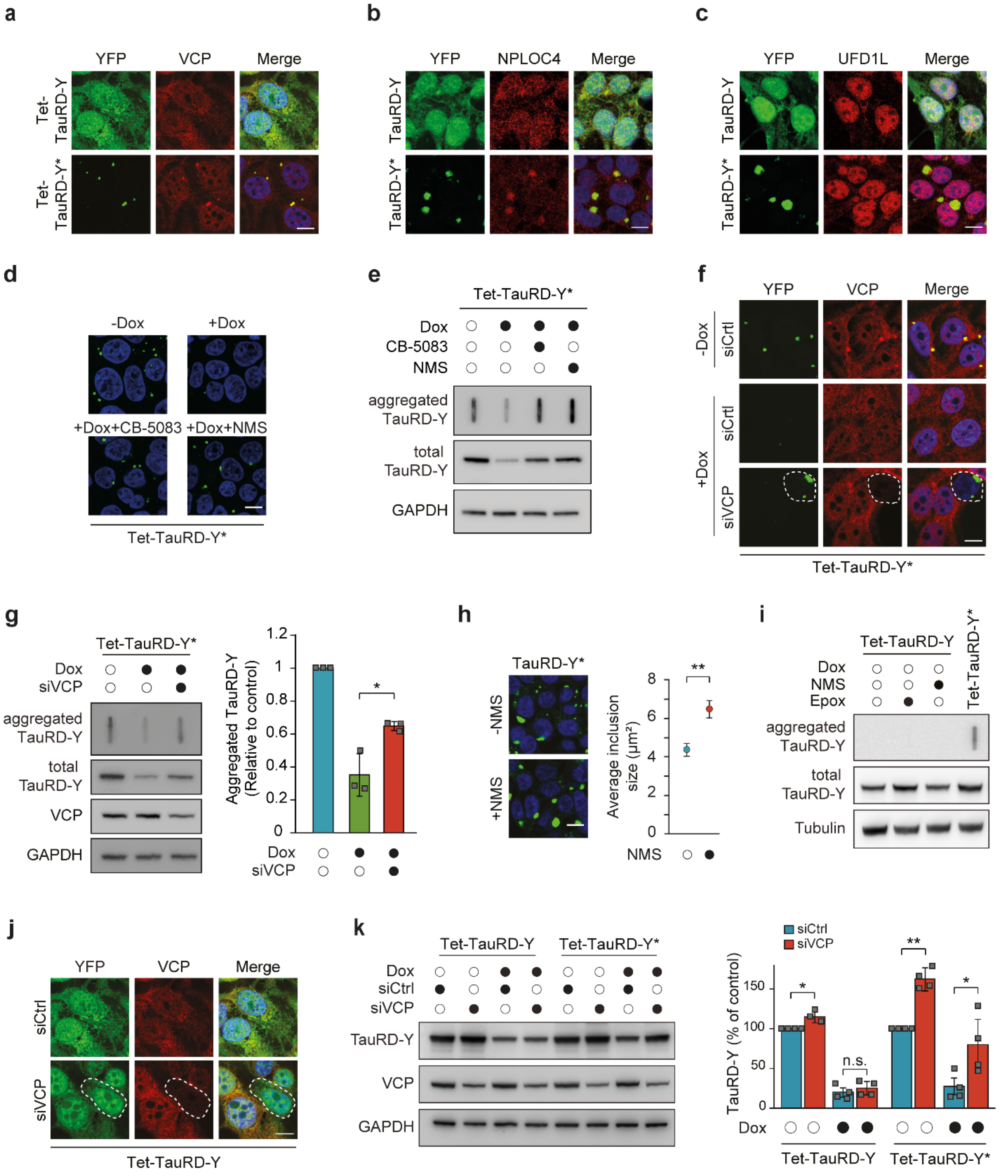

Supplementary Fig. 5: Aggregation specific stabilization of Tau by VCP inactivation.

a Immunofluorescence staining of VCP (red) and YFP fluorescence of TauRD-Y (green). **b** and **c** Immunofluorescence staining of NPLOC4 (b) (red) and UFD1L (c) (red). Scale bars, 10 μm. **d** Representative images of Tet-TauRD-Y* cells treated with doxycycline (Dox; 50 ng/mL) alone

or in combination with CB-5083 (1 μM) or NMS-873 (NMS; 2.5 μM). Scale bar, 10 μm. **e** Filter trap analysis of Tet-TauRD-Y* cells treated as in (d). **f** Immunofluorescence staining of VCP (red) in Tet-TauRD-Y* cells treated with non-targeted (Ctrl) or VCP siRNA for 96 h. Doxycycline (Dox; 50 ng/mL) was added for the last 24 h. Dashed lines indicate a cell with reduced VCP levels. Scale bar, 10 μm. **g** Left, filter trap analysis of aggregated TauRD-Y in Tet-TauRD-Y* lysates treated as in (f). Right, quantification of aggregated TauRD-Y. Mean ± s.d.; n=3. *p<0.05 (p= 0.0174) from two-tailed Student's paired t-test. **h** Representative images of TauRD-Y* cells treated with NMS-873 (NMS; 5 μM) and quantification of average inclusion size (μm^2). 200-400 cells analyzed per experiment. Mean ± s.d.; n=5. **p<0.01 (p= 0.0022) from two-tailed Student's paired t-test. **i** Filter trap analysis of Tet-TauRD-Y cells treated with Epoxomicin (Epox; 50 nM) or NMS-873 (NMS; 2.5 μM). Tet-TauRD-Y* lysate was used as control. **j** Immunofluorescence staining of VCP (red) and YFP fluorescence of TauRD-Y (green) in Tet-TauRD-Y cells transfected with non-targeted (Ctrl) or VCP siRNA. Dashed lines indicate a cell with reduced VCP levels. Scale bar, 10 μm. **k** Left, analysis of TauRD-Y level in Tet-TauRD-Y and Tet-TauRD-Y* cells transfected for 96 h with non-targeted (Ctrl) or VCP siRNA. Doxycycline (Dox; 50 ng/mL) was added for the last 24 h. TauRD-Y was detected with anti-TauRD antibody. Right, quantification of TauRD-Y immunoblot. Mean ± s.d.; n=4. *p<0.05 (Tet-TauRD-Y - Dox: siCtrl vs siVCP, p= 0.0218; Tet-TauRD-Y* + Dox: siCtrl vs siVCP, p= 0.0156); **p<0.01 (Tet-TauRD-Y* - Dox: siCtrl vs siVCP, p= 0.0023); n.s. non-significant (Tet-TauRD-Y + Dox: siCtrl vs siVCP, p= 0.0539) from two-tailed paired Student's t-test. Source data are provided as a Source Data file.

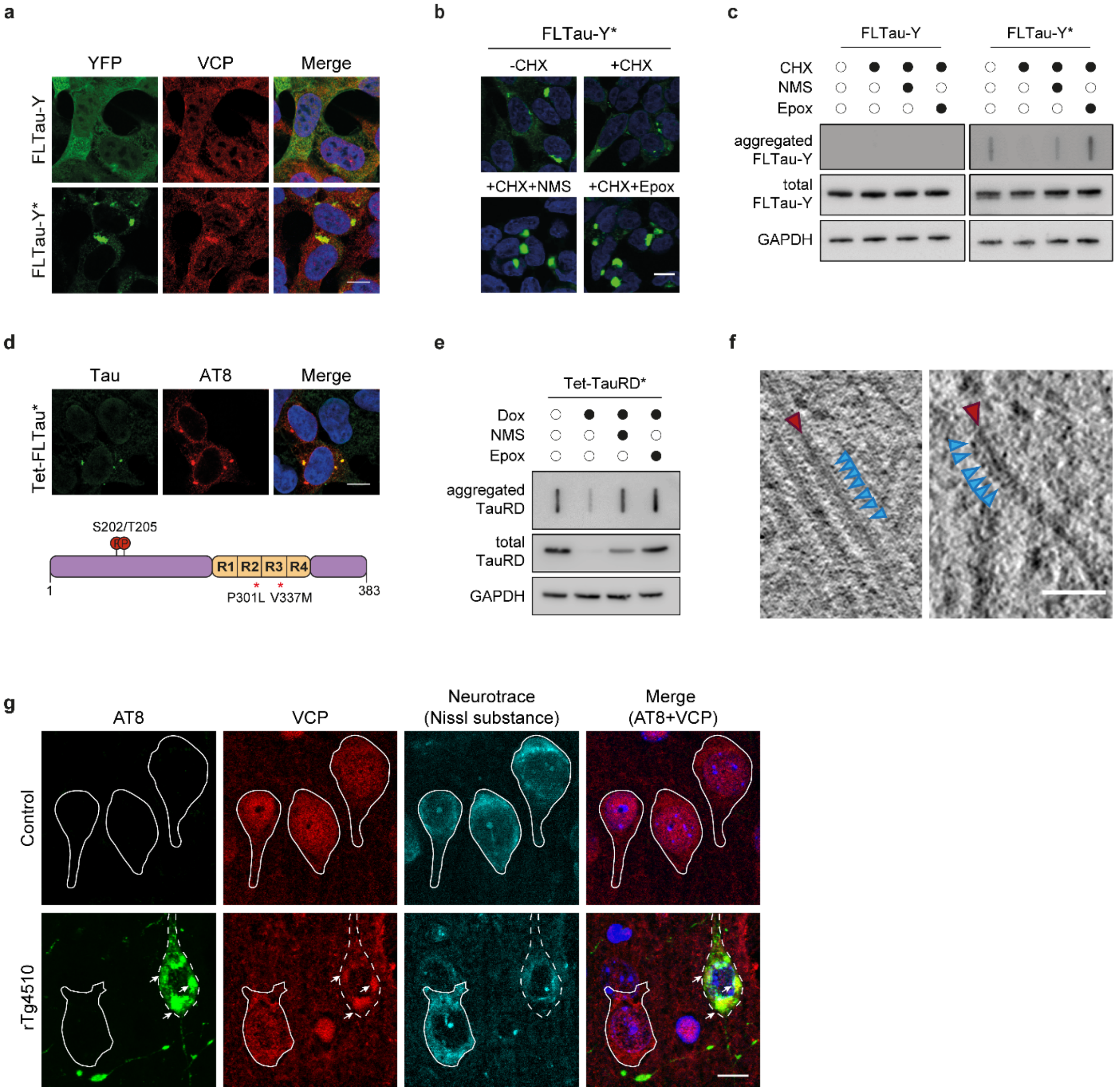

Supplementary Fig. 6: Role of VCP in full length Tau disaggregation.

a Immunofluorescence staining of VCP (red) and YFP fluorescence of FLTau-Y (green) in FLTau-Y and FLTau-Y* cells. Scale bar, 10 µm. **b** Representative images of FLTau-Y* cells treated for 24 h with cycloheximide (CHX; 50 µg/mL) alone or in combination with NMS-873 (NMS; 2.5 µM) or Epoxomicin (Epox; 100 nM). Scale bar, 10 µm. **c** Filter trap analysis of lysates from FLTau-Y and FLTau-Y* cells treated for 24 h with Dox alone or in combination with NMS-873 (NMS; 2.5 µM) or Epoxomicin (Epox; 50 nM). Aggregated and total FLTau-Y levels were

determined by immunoblotting against GFP. GAPDH served as loading control. **d** Immunofluorescence staining of full-length Tau (FLTau) in aggregate-containing Tet-FLTau* cells with Tau (green) and Tau S202/T205 phosphorylation specific AT-8 (red) antibody. Scale bar, 10 μm. **e** Filter trap analysis of lysates from Tet-TauRD* cells treated for 24 h with Dox alone or in combination with NMS-873 (NMS; 2.5 μM) or Epoxomicin (Epox; 50 nM). Aggregated and total TauRD levels were determined by immunoblotting against myc and TauRD, respectively. GAPDH served as loading control. **f** Examples of two TauRD-Y fibrils from a representative 1.4 nm thick tomographic slice of a TauRD inclusion from neurons. Red arrowheads indicate TauRD-Y fibrils and blue arrowheads indicate globular densities along fibrils. Scale bar, 40 nm. **g** Immunofluorescence staining of brain sections of 4-month-old control and Tau transgenic rTg4510 mice with AT8 (green) and VCP (red) antibodies, Nissl substance (cyan) and DAPI (blue in the merged image). The outline of a cell containing phosphorylated Tau (p-Tau) is marked by a white dashed line. Cells not containing detectable p-Tau are marked by white continuous lines. Arrows point to VCP colocalizing with Tau inclusions. Scale bar, 10 μm.

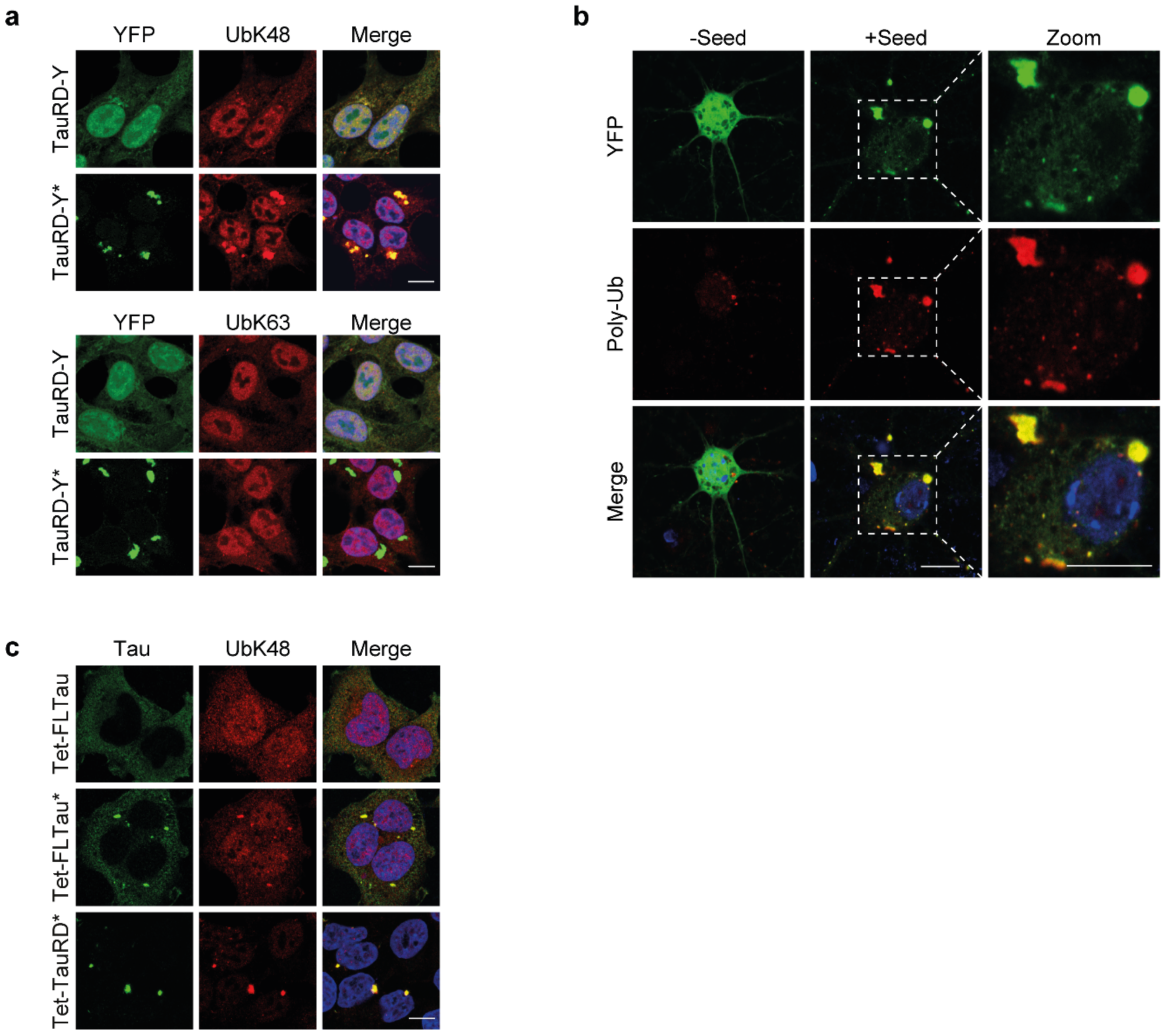

Supplementary Fig. 7: Ubiquitylation of TauRD-Y aggregates.

a Immunofluorescence staining of (top) ubiquitin-K48 (UbK48) (red) and (bottom) ubiquitin K63 (UbK63) (red) chains and YFP fluorescence of TauRD-Y (green) in TauRD-Y and TauRD-Y* cells. Scale bars, 10 µm. **b** Immunofluorescence staining of ubiquitylated proteins (FK2 antibody) (red) in primary neurons expressing TauRD-Y (green) and treated with TauRD containing lysates (+Seed) where indicated. Scale bars, 20 µm. **c** Immunofluorescence staining of ubiquitin-K48 (UbK48) (red) chains and Tau (green) in Tet-FLTau, Tet-FLTau* and Tet-TauRD* cells. FLTau was detected using Tau-5 and TauRD using anti-myc antibody. Scale bar, 10 µm.

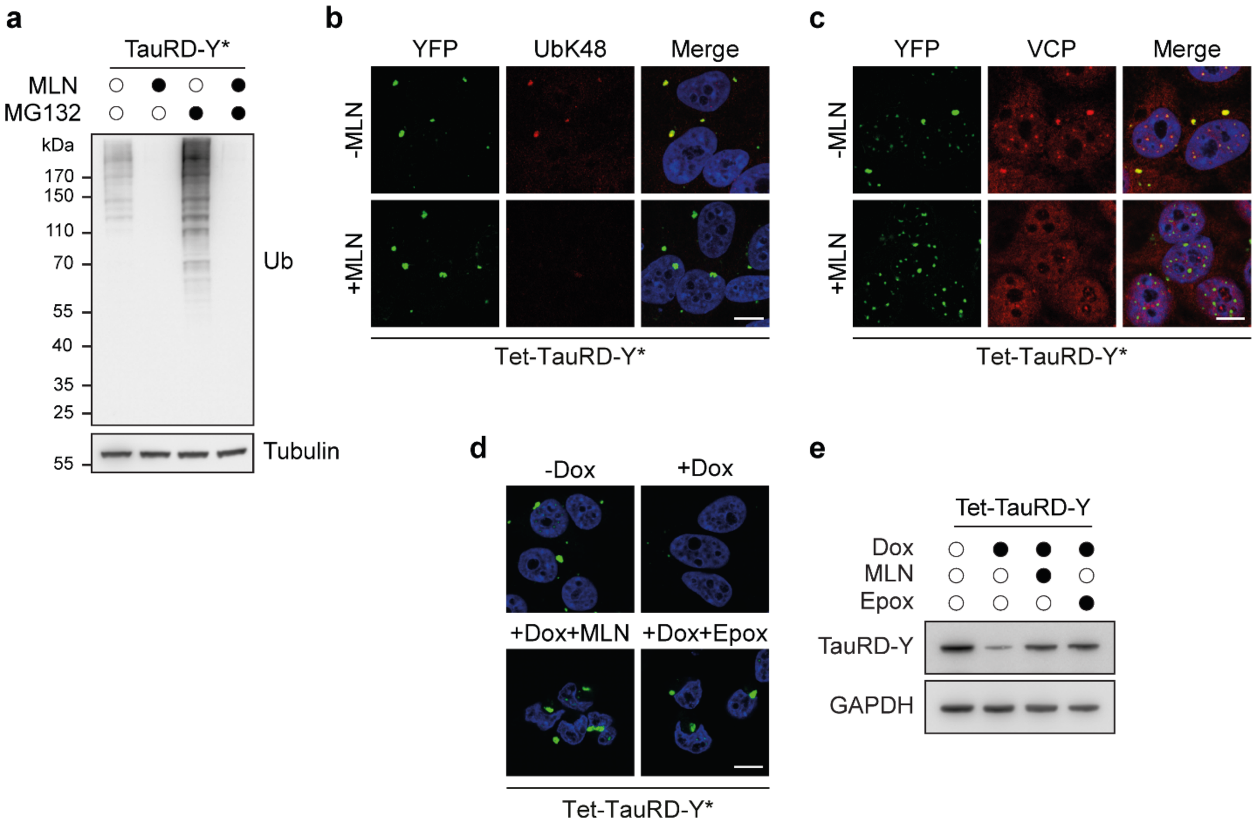

Supplementary Fig. 8: Role of ubiquitylation in TauRD-Y disaggregation.

a Analysis of ubiquitylated protein levels in lysates of TauRD-Y* cells treated with the ubiquitin activating enzyme E1 inhibitor MLN7243 (MLN; 0.5 µM) alone or in combination with proteasome inhibitor MG132 (1 µM) for 14 h. Ubiquitylated proteins were detected by immunoblotting against ubiquitin. Tubulin served as loading control. **b** Immunofluorescence staining of ubiquitin-K48 chains (UbK48) (red) and **c** VCP (red) in Tet-TauRD-Y* cells treated with MLN7243 (MLN; 0.5 µM) for 12 h. Scale bars, 10 µm. **d** Representative images of Tet-TauRD-Y* cells treated for 24 h with doxycycline (Dox; 50 ng/mL) alone or in combination with MLN7243 (MLN; 0.5 µM) or Epoxomicin (Epox; 50 nM). Scale bar, 10 µm. **e** Analysis of TauRD-Y levels in Tet-TauRD-Y cells treated for 24 h with Dox, MLN7243 and Epoxomicin as in (d). GAPDH served as loading control.

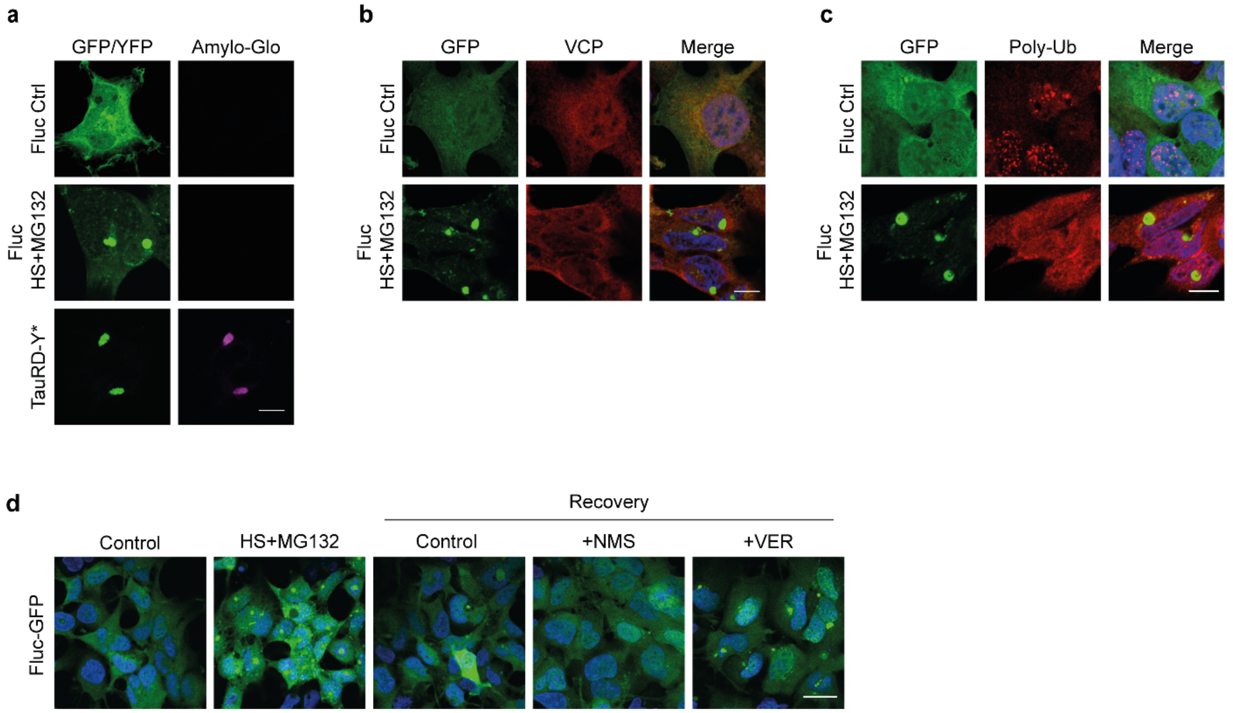

Supplementary Fig. 9: Effect of VCP inhibition on firefly luciferase (Fluc) disaggregation.

a Fluc-GFP expressing cells maintained at 37 °C (Fluc Ctrl) or heat-stressed at 43 °C in presence of 5 µM MG132 for 2 h (Fluc HS) were stained with the amyloid-specific dye Amylo-Glo (magenta). TauRD-Y* cells were used as control. Amylo-Glo fluorescence was imaged with similar exposure settings in all panels. Scale bar, 10 µm. **b** Immunofluorescence staining of VCP (red), and **c** ubiquitylated proteins (FK2 antibody) (red) in Fluc-GFP cells treated as in (a). Scale bars, 10 µm. **d** Effect of VCP and Hsp70 inhibition on Fluc-GFP disaggregation. Fluc-GFP aggregation was induced as in (a). Cells were then shifted to MG132 free media and allowed to recover at 37 °C for 8 h in presence of NMS-873 (NMS; 2.5 µM) and VER-155008 (VER; 10 µM) where indicated. Scale bar, 30 µm.

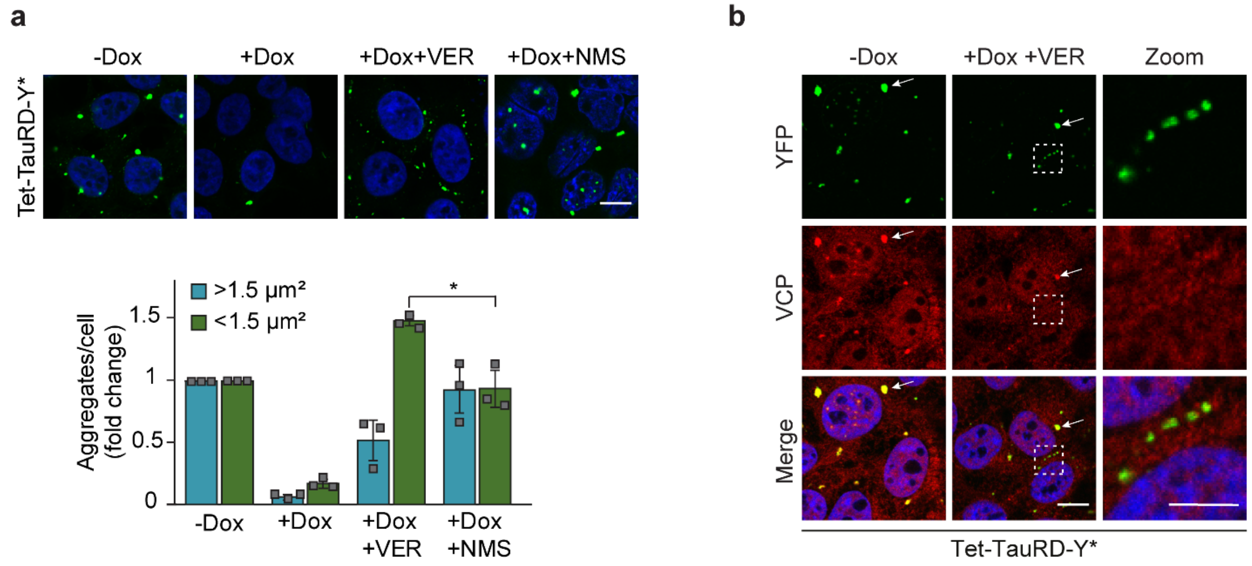

Supplementary Fig. 10: Role of Hsp70 in TauRD-Y disaggregation.

a Top, Representative images of Tet-TauRD-Y* cells treated for 24 h with doxycycline (Dox; 50 ng/mL) alone or in combination with VER-155008 (VER; 10 µM) or NMS-873 (NMS; 2.5 µM). Bottom, quantification of large (>1.5 μm^2) and small (<1.5 μm^2) TauRD-Y foci. Mean ± s.d.; n=3; ~100-200 cells were analyzed per experiment. *p<0.05 (p=0.0435) from two-tailed Student's paired t-test. Scale bar, 10 µm. **b** Immunofluorescence staining of VCP (red) and YFP fluorescence of TauRD-Y (green) in Tet-TauRD-Y* cells treated with a combination of doxycycline (Dox) and VER-155008 (VER) where indicated. White arrow points to large TauRD-Y inclusions co-localizing with VCP. Dashed lines enclose TauRD-Y foci that do not co-localize with VCP. Scale bar, 10 µm. Scale bar zoom, 5 µm. Source data are provided as a Source Data file.

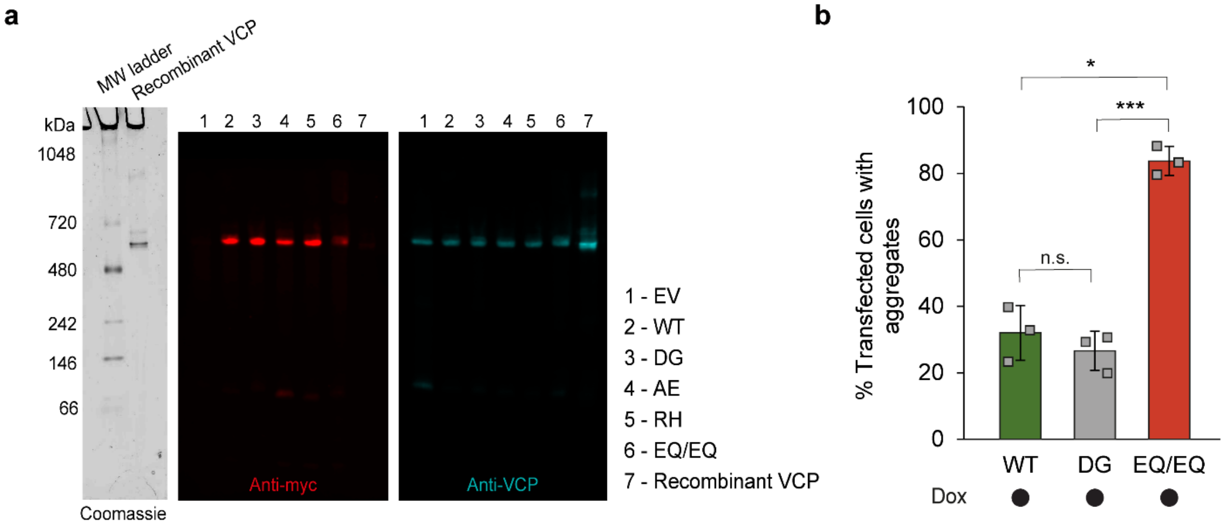

Supplementary Fig. 11: Effect of VCP mutants on Tau disaggregation.

a Native-PAGE analysis of recombinant VCP and lysates from Tet-TauRD-Y* cells transfected with empty vector (EV) and myc-tagged wild type (WT), D395G (DG), A232E (AE), R155H (RH) and E305Q/E578Q (EQ/EQ) VCP constructs. Immunoblot probed against myc (red) and VCP (cyan) is shown. Non-tagged, recombinant VCP was analyzed as control. **b** Quantification of aggregate foci in myc-positive Tet-TauRD-Y* cells transfected with myc-tagged WT, DG and EQ/EQ VCP constructs for 24 h, and treated for another 24 h with doxycycline (Dox; 50 ng/mL). Mean ± s.d.; n=3; > 100 cells analyzed per experiment; *$p<0.05$ (WT vs EQ/EQ $p=0.0192$); ***$p<0.001$ (DG vs EQ/EQ $p=0.0008$); n.s. non-significant ($p=0.5646$). Source data are provided as a Source Data file.

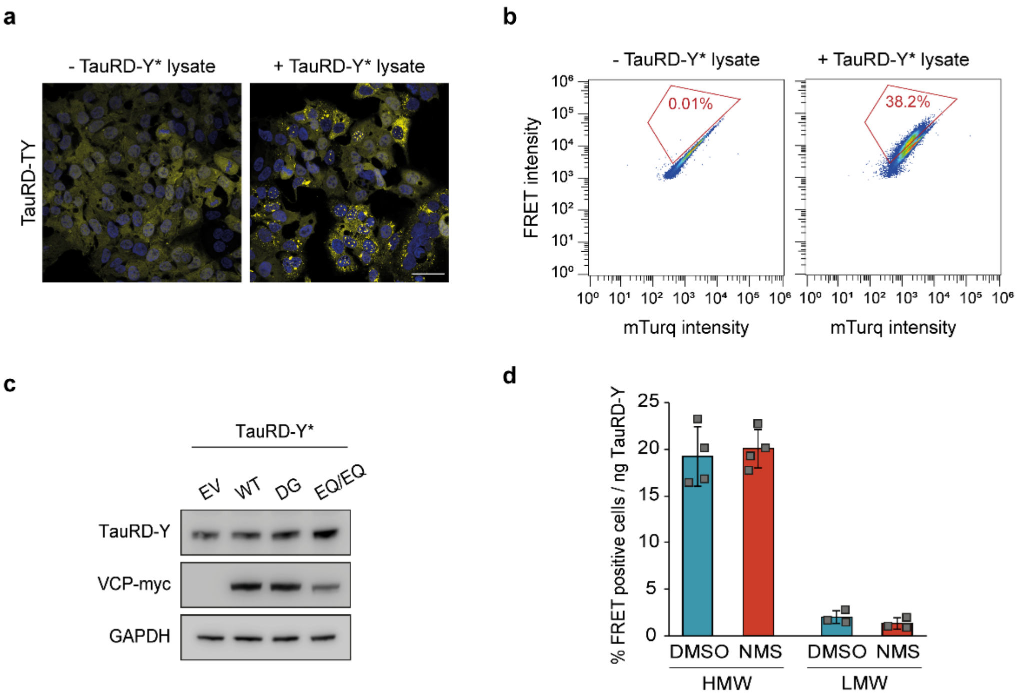

Supplementary Fig. 12: Analysis of seeding-competent TauRD-Y.

a Representative images of TauRD-TY FRET reporter cells treated with TauRD-Y* lysate where indicated showing TauRD-Y florescence in yellow. Scale bar, 40 µm. **b** Representative pseudocolour dot plots for the analysis of FRET positive TauRD-TY cells by flow cytometry upon addition of TauRD-Y* lysate. FRET intensity is plotted against mTurquoise2 (mTurq) intensity and the % of FRET positive cells are indicated in red gates. **c** Analysis of TauRD-Y and VCP-myc levels in TauRD-Y* cells transfected for two days with empty vector (EV) and myc-tagged wild type (WT), D395G (DG) and E305Q/E578Q (EQ/EQ) VCP constructs. TauRD-Y and overexpressed VCP levels were determined by immunoblotting against GFP and myc, respectively. GAPDH served as loading control. **d** Comparison of seeding efficiencies of high molecular weight (HMW) and low molecular weight (LMW) species obtained by size exclusion chromatography of lysates from TauRD-Y* cells treated for 24 h with DMSO or NMS-873 (NMS; 2 µM). Mean ± s.d.; HMW n=4, LMW n=3. Source data are provided as a Source Data file.

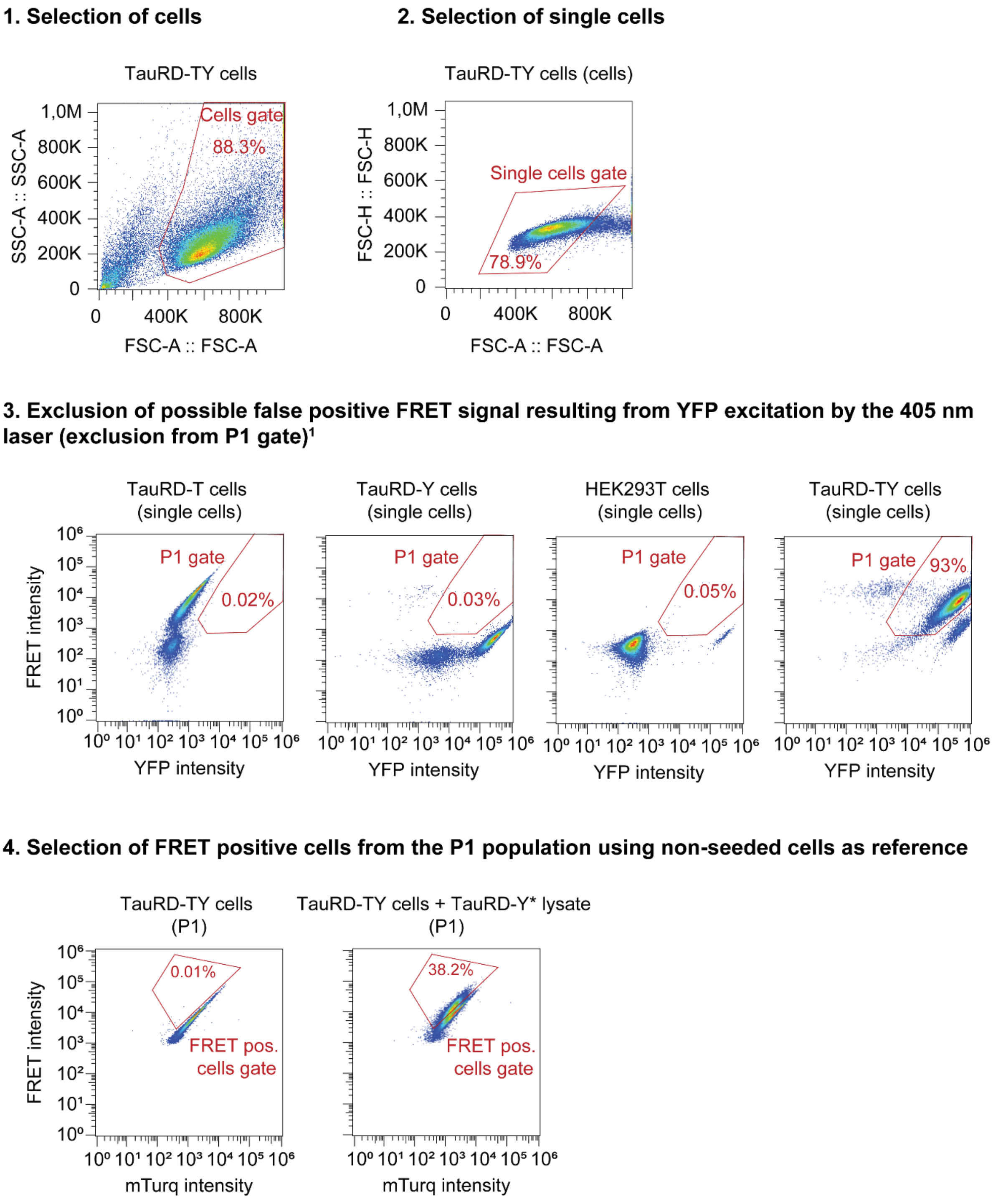

Supplementary Fig. 13: Flow cytometry strategy for quantification of FRET positive cells. To measure the mTurquoise2 and FRET fluorescence signals, cells were excited with 405 nm laser light and fluorescence was determined using 440/50 and 530/30 filters, respectively. To measure the YFP fluorescence signal, cells were excited at 488 nm and emission was recorded using a

530/30 filter. For each sample, 50,000 single cells were analyzed, using a gating strategy recently described[1,2]. First, cells were selected (1), followed by single cell selection (2). After gating single cells, an additional gate (P1) was introduced to exclude YFP-only cells that show a false-positive signal in the FRET channel due to excitation at 405 nm using TauRD-T cells, TauRD-Y cells and HEK293T cells[1,2] as reference (3). The FRET positive gate was set by plotting the FRET fluorescence signal versus the mTurquoise2 fluorescence signal using non-seeded cells as reference (4). Note that some panels in (1)-(3) were recently published describing the gating strategy for a comparable experiment[2].

Supplementary references

1 Banning, C. *et al.* A flow cytometry-based FRET assay to identify and analyse protein-protein interactions in living cells. *PLoS One* **5**, e9344 (2010).

2 Yuste-Checa, P. *et al.* The extracellular chaperone Clusterin enhances Tau aggregate seeding in a cellular model. *Nature Communications* **12**, 4863 (2021).

Supplementary Table 1. TauRD-Y interactome in TauRD-Y* cells

List of proteins interacting with TauRD-Y in TauRD-Y* cells at steady state determined by stable isotope labelling by amino acids in cell culture (SILAC). TauRD-Y cells were used as control. Normalized SILAC ratios of TauRD-Y*/TauRD-Y [H/L] from 3 independent replicates are shown. Interactors were defined as proteins quantified in at least 2 out of 3 replicates with enrichment ≥2 fold. Proteins with known association to the ubiquitin-proteasome system are highlighted in green and VCP-cofactor complex in red. Intensity-based absolute quantification (iBAQ) values reflect measured molar protein amounts.

Protein ID	Protein name	Gene name	Fasta headers	Unique peptides	Mol. weight [kDa]	Norm. ratio [H/L] Rep.1	Norm. ratio [H/L] Rep.2	Norm. ratio [H/L] Rep.3	Median norm. ratio	iBAQ [H] Exp.1	iBAQ [H] Exp.2	iBAQ [H] Exp.3
Q9UNZ2;F2Z	NSFL1 cofactor p47	NSFL1C	sp|Q9UNZ2|NSF1C_HUMAN NSFL1 cofactor p47 OS	14	40.572	42.702	80.022	11.719	42.702	59143000	112180000	13783000
Q9UQN3;A0	Charged multivesicular body protein 2b	CHMP2B	sp|Q9UQN3|CHM2B_HUMAN Charged multivesicul	4	23.906	18.201	25.514	11.839	18.201	2093000	5417800	15472000
P55072;C9IZ	Transitional endoplasmic reticulum ATPase	VCP	sp|P55072|TERA_HUMAN Transitional endoplasmi	47	89.321	16.959	43.344	6.3181	16.959	29193000	94165000	26411000
Q9UNM6;J3	26S proteasome non-ATPase regulatory sub	PSMD13	sp|Q9UNM6|PSD13_HUMAN 26S proteasome non-	22	42.945	17.963	16.071	13.583	16.071	9313200	17290000	5085700
Q6PEV8	Protein FAM199X	FAM199X	sp|Q6PEV8|F199X_HUMAN Protein FAM199X OS=H	5	42.801	41.449	15.154	15.115	15.154	9838300	30031000	10267000
F8VUA2;Q9	Charged multivesicular body protein 1a	CHMP1A	tr|F8VUA2|F8VUA2_HUMAN Charged multivesicul	5	19.531	14.648	19.186	12.293	14.648	25912000	113020000	38592000
Q15773;F5H	Myeloid leukemia factor 2	MLF2	sp|Q15773|MLF2_HUMAN Myeloid leukemia facto	6	28.147	16.867	13.819	6.564	13.819	74907000	153440000	42871000
O43242;H0Y	26S proteasome non-ATPase regulatory sub	PSMD3	sp|O43242|PSMD3_HUMAN 26S proteasome non-A	34	60.977	13.413	15.879	6.9978	13.413	21734000	50167000	10257000
O00231;J3Q	26S proteasome non-ATPase regulatory sub	PSMD11	sp|O00231|PSD11_HUMAN 26S proteasome non-A	26	47.463	13.086	22.385	7.5615	13.086	14133000	35102000	7362100
P62191;G3V	26S protease regulatory subunit 4	PSMC1	sp|P62191|PRS4_HUMAN 26S proteasome regulato	20	49.184	12.739	13.685	6.7346	12.739	6082800	18968000	4723500
P17480	Nucleolar transcription factor 1	UBTF	sp|P17480|UBF1_HUMAN Nucleolar transcription f	22	89.405	12.209	11.585	7.1602	11.585	94071000	144030000	43389000
O14545;F8V	TRAF-type zinc finger domain-containing pr	TRAFD1	sp|O14545|TRAD1_HUMAN TRAF-type zinc finger d	12	64.84	7.2319	19.2	11.522	11.522	6914300	16022000	16301000
Q13501;E7E	Sequestosome-1	SQSTM1	sp|Q13501|SQSTM_HUMAN Sequestosome-1 OS=H	16	47.687	9.6152	17.975	11.41	11.41	27933000	113830000	115270000
Q13200;C9J	26S proteasome non-ATPase regulatory sub	PSMD2	sp|Q13200|PSMD2_HUMAN 26S proteasome non-A	46	100.2	7.2067	22.927	11.267	11.267	4555900	9942100	6380700
H0Y6K2;A0A	Bromodomain-containing protein 2	BRD2	tr|H0Y6K2|H0Y6K2_HUMAN Bromodomain-contain	11	88.288	24.922	7.6855	10.958	10.958	6685300	16169000	6613700
H0YFD6;P40	Trifunctional enzyme subunit alpha, mitoch	HADHA	tr|H0YFD6|H0YFD6_HUMAN Trifunctional enzyme s	29	86.371	10.902	11.422	8.175	10.902	45516000	92103000	31426000
P55036;Q5V	26S proteasome non-ATPase regulatory sub	PSMD4	sp|P55036|PSMD4_HUMAN 26S proteasome non-A	14	40.736	12.228	10.9	3.7986	10.9	19080000	82099000	8516700
Q6QNY1;J3C	Biogenesis of lysosome-related organelles	BLOC1S2	sp|Q6QNY1|BL1S2_HUMAN Biogenesis of lysosom	3	15.961	10.78	15.848	2.0051	10.78	1892700	3652000	1718300
P55084;F5G	Trifunctional enzyme subunit beta, mitocho	HADHB	sp|P55084|ECHB_HUMAN Trifunctional enzyme su	25	51.294	9.9693	11.079	8.0079	9.9693	65103000	128900000	39724000
P25788;G3V	Proteasome subunit alpha type-3	PSMA3	sp|P25788|PSA3_HUMAN Proteasome subunit alph	9	28.433	3.0605	14.754	9.9671	9.9671	2846500	1743900	2866700
A8MUA9;A8	Small ubiquitin-related modifier 4;Small ub	SUMO3;SUM	tr|A8MUA9|A8MUA9_HUMAN SMT3 suppressor of	1	15.317	8.4377	11.126		9.78185	14432000	26087000	0
Q8TAT6;I3L	Nuclear protein localization protein 4 homo	NPLOC4	sp|Q8TAT6|NPL4_HUMAN Nuclear protein localiza	17	68.119	3.9291	15.451		9.69005	1208400	5604000	0
P25789;H0Y	Proteasome subunit alpha type-4;Proteasor	PSMA4	sp|P25789|PSA4_HUMAN Proteasome subunit alph	10	29.483	2.7706		16.307	9.5388	4339400	0	4387000
Q9UHD9	Ubiquilin-2	UBQLN2	sp|Q9UHD9|UBQL2_HUMAN Ubiquilin-2 OS=Homo	11	65.695	6.6843	11.794		9.23915	8201000	24600000	108990
O14818;H0Y	Proteasome subunit alpha type-7	PSMA7	sp|O14818|PSA7_HUMAN Proteasome subunit alp	14	27.887	2.6045	9.1585	10.57	9.1585	3845500	10551000	10021000
Q9UID3;E9P	Vacuolar protein sorting-associated protein	VPS51	sp|Q9UID3|VPS51_HUMAN Vacuolar protein sortin	11	86.041	12.8	5.2875		9.04375	603160	3513600	552200
Q14596;B7Z	Next to BRCA1 gene 1 protein	NBR1	sp|Q14596|NBR1_HUMAN Next to BRCA1 gene 1 pr	12	107.41		15.113	2.4528	8.7829	0	12693000	12934000
Q92890;C9J	Ubiquitin fusion degradation protein 1 hom	UFD1L	sp|Q92890|UFD1_HUMAN Ubiquitin recognition fa	9	34.5	11.411	6.134		8.7725	785590	5835400	721170
Q15008;C9J	26S proteasome non-ATPase regulatory sub	PSMD6	sp|Q15008|PSMD6_HUMAN 26S proteasome non-A	27	45.531	7.3639	9.6008	8.555	8.555	13494000	32338000	6986900

Continued on next page

Protein ID	Protein name	Gene name	Fasta headers	Unique peptides	Mol. weight [kDa]	Norm. ratio [H/L] Rep.1	Norm. ratio [H/L] Rep.2	Norm. ratio [H/L] Rep.3	Median norm. ratio	iBAQ [H] Exp.1	iBAQ [H] Exp.2	iBAQ [H] Exp.3
Q96DX7	Tripartite motif-containing protein 44	TRIM44	sp|Q96DX7|TRI44_HUMAN Tripartite motif-contair	5	38.472	10.369	6.5983		8.48365	708170	19187000	783500
Q16643;D6F	Drebrin	DBN1	sp|Q16643|DREB_HUMAN Drebrin OS=Homo sapie	16	71.428		13.93	2.7427	8.33635	123350	13631000	6342500
A0A087X2I1	26S protease regulatory subunit 10B	PSMC6	tr|A0A087X2I1|A0A087X2I1_HUMAN 26S proteasor	16	45.796	10.533	6.0398		8.2864	2266900	10434000	743130
P51665;H3B	26S proteasome non-ATPase regulatory sub	PSMD7	sp|P51665|PSMD7_HUMAN 26S proteasome non-A	14	37.025	8.2466	13.713	6.6956	8.2466	9133800	34517000	6064400
P35998;A0A	26S protease regulatory subunit 7	PSMC2	sp|P35998|PRS7_HUMAN 26S proteasome regulatc	24	48.633	7.5017	8.0742	8.5178	8.0742	8531200	12549000	5021100
B3KVL5;Q8T	Zinc finger CCHC domain-containing proteir	ZCCHC10	tr|B3KVL5|B3KVL5_HUMAN Zinc finger, CCHC dom	1	20.308		7.1775	8.4956	7.83655	18527000	60508000	11466000
Q8IXW5	Putative RNA polymerase II subunit B1 CTD	RPAP2	sp|Q8IXW5|RPAP2_HUMAN Putative RNA polymer	13	69.508	10.475	7.8181	3.3996	7.8181	1205400	8566200	4524400
P62979;J3Q	Ubiquitin-40S ribosomal protein S27a;Ubiqu	RPS27A;UBB;	sp|P62979|RS27A_HUMAN Ubiquitin-40S ribosoma	10	17.965	9.4683	7.6945	4.1911	7.6945	3164100000	7019100000	1951400000
Q99460;A0A	26S proteasome non-ATPase regulatory sub	PSMD1	sp|Q99460|PSMD1_HUMAN 26S proteasome non-A	42	105.84	6.684	7.415	7.8358	7.415	3058500	12531000	2851500
P43686	26S protease regulatory subunit 6B	PSMC4	sp|P43686|PRS6B_HUMAN 26S proteasome regulat	21	47.366	8.4576	2.5541	6.7907	6.7907	1137200	10458000	617140
R4GMR5;K7	26S proteasome non-ATPase regulatory sub	PSMD8	tr|R4GMR5|R4GMR5_HUMAN 26S proteasome non	10	32.551	1.3779	6.6145	13.37	6.6145	491820	3751000	6211100
P0CAP2;H8Y	DNA-directed RNA polymerase II subunit GF	POLR2M;GCC	sp|P0CAP2|GRL1A_HUMAN DNA-directed RNA pol	4	41.739	6.9552	6.1579		6.55655	2999600	6478700	0
P49721;A0A	Proteasome subunit beta type-2	PSMB2	sp|P49721|PSB2_HUMAN Proteasome subunit beta	10	22.836	2.6138	10.075		6.3444	526870	3115400	1949400
Q9UQ35;I3L	Serine/arginine repetitive matrix protein 2	SRRM2	sp|Q9UQ35|SRRM2_HUMAN Serine/arginine repet	44	299.61	8.8182	4.1061	6.3418	6.3418	40059000	69443000	34477000
O75487	Glypican-4;Secreted glypican-4	GPC4	sp|O75487|GPC4_HUMAN Glypican-4 OS=Homo sa	13	62.411	6.1176	9.697	5.2264	6.1176	12306000	31449000	4359700
Q9H307;G3V	Pinin	PNN	sp|Q9H307|PININ_HUMAN Pinin OS=Homo sapien	39	81.627	6.4613	5.9829	3.8631	5.9829	180190000	359840000	205880000
Q99615;K7E	DnaJ homolog subfamily C member 7	DNAJC7	sp|Q99615|DNJC7_HUMAN DnaJ homolog subfami	35	56.44	32.017	5.9433	3.8506	5.9433	58381000	56886000	13723000
O95816	BAG family molecular chaperone regulator	BAG2	sp|O95816|BAG2_HUMAN BAG family molecular ch	9	23.772	5.7371	8.5666	3.8905	5.7371	10758000	36658000	8833600
Q9BYN8	28S ribosomal protein S26, mitochondrial	MRPS26	sp|Q9BYN8|RT26_HUMAN 28S ribosomal protein S	7	24.211	5.6963	5.8954	3.1697	5.6963	15879000	55778000	11868000
P10644;K7E	cAMP-dependent protein kinase type I-alph	PRKAR1A	sp|P10644|KAP0_HUMAN cAMP-dependent protei	12	42.981	5.6698	8.1797	2.4289	5.6698	1804900	8873600	3456600
A0A0C4DG6	ADP-ribosylation factor-like protein 6-inter	ARL6IP4	tr|A0A0C4DG62|A0A0C4DG62_HUMAN ADP-ribosy	5	24.591	5.6534	5.8168	2.4357	5.6534	11791000	37307000	7853000
Q15545	Transcription initiation factor TFIID subunit	TAF7	sp|Q15545|TAF7_HUMAN Transcription initiation f	5	40.259	6.7383	4.5475		5.6429	1917000	3824800	262590
F5H442;Q9	Tumor susceptibility gene 101 protein	TSG101	tr|F5H442|F5H442_HUMAN Tumor susceptibility ge	8	40.917	6.9756	3.423		5.1993	1298100	2512100	0
Q14677;H0Y	Clathrin interactor 1	CLINT1	sp|Q14677|EPN4_HUMAN Clathrin interactor 1 OS=	17	68.259	4.5671	5.1051		4.8361	483270	1390800	0
Q16531;F5G	DNA damage-binding protein 1	DDB1	sp|Q16531|DDB1_HUMAN DNA damage-binding pr	51	126.97	5.1552	4.8124	4.7395	4.8124	2260500	2015000	818320
O14646;H0Y	Chromodomain-helicase-DNA-binding prot	CHD1	sp|O14646|CHD1_HUMAN Chromodomain-helicas	22	196.69	4.667	5.6909	3.5182	4.667	1212300	6836700	855680
G3V5Z7;P60	Proteasome subunit alpha type;Proteasome	PSMA6	tr|G3V5Z7|G3V5Z7_HUMAN Proteasome subunit a	12	28.147		5.4361	3.7845	4.6103	0	986560	3211200
P17980;E9P	26S protease regulatory subunit 6A	PSMC3	sp|P17980|PRS6A_HUMAN 26S proteasome regula	21	49.203	5.2382	3.8803		4.55925	2297800	7983700	563940
O00232;J3K	26S proteasome non-ATPase regulatory sub	PSMD12	sp|O00232|PSD12_HUMAN 26S proteasome non-A	22	52.904	4.4642	2.2919	13.215	4.4642	5157500	5534500	3641700
P61964;V9G	WD repeat-containing protein 5	WDR5	sp|P61964|WDR5_HUMAN WD repeat-containing p	5	36.588	2.7665	5.5202		4.14335	1116200	6218500	163100
O00487;C9J	26S proteasome non-ATPase regulatory sub	PSMD14	sp|O00487|PSDE_HUMAN 26S proteasome non-AT	10	34.577	4.4008	3.6454		4.0231	2763600	4175800	1273000
P62195;J3Q	26S protease regulatory subunit 8	PSMC5	sp|P62195|PRS8_HUMAN 26S proteasome regulatc	23	45.626	4.5333	4.0178	2.4837	4.0178	2401900	10162000	3258800
P24928	DNA-directed RNA polymerase II subunit RF	POLR2A	sp|P24928|RPB1_HUMAN DNA-directed RNA polyr	51	217.17	3.9675	4.1625	3.3865	3.9675	5036400	22719000	4400000
O14974;F8V	Protein phosphatase 1 regulatory subunit 1	PPP1R12A	sp|O14974|MYPT1_HUMAN Protein phosphatase 1	15	115.28	3.6323	4.1458		3.88905	135210	738040	0
Q5VIR6;F6V	Vacuolar protein sorting-associated protein	VPS53	sp|Q5VIR6|VPS53_HUMAN Vacuolar protein sortin	10	79.652		2.3872	5.2434	3.8153	0	867750	158630
Q8WV44;H0	E3 ubiquitin-protein ligase TRIM41	TRIM41	sp|Q8WV44|TRI41_HUMAN E3 ubiquitin-protein li	5	71.669	4.3208	3.4885	3.7734	3.7734	613000	252640	1261800
F8W118;F8VV59;B7Z9C2;F8VRJ2		NAP1L1	tr|F8W118|F8W118_HUMAN Nucleosome assembl	12	24.694	3.6447	4.8952	3.4073	3.6447	4807500	20158000	2227100
E9PNW4;A0	CD59 glycoprotein	CD59	tr|E9PNW4|E9PNW4_HUMAN Uncharacterized pro	4	11.985		4.7462	2.1357	3.44095	5131200	54954000	35045000
Q96BQ5	Coiled-coil domain-containing protein 127	CCDC127	sp|Q96BQ5|CC127_HUMAN Coiled-coil domain-co	4	30.834	4.0401		2.7807	3.4104	3768100	3178900	697410
O43164	E3 ubiquitin-protein ligase Praja-2	PJA2	sp|O43164|PJA2_HUMAN E3 ubiquitin-protein liga	15	78.213	3.3397	6.1296	2.2392	3.3397	4791500	29710000	8235700
B8ZZD4;Q8	Tax1-binding protein 1	TAX1BP1	tr|B8ZZD4|B8ZZD4_HUMAN Tax1-binding protein 1	14	93.609	9.5213	3.2831	3.1608	3.2831	409770	4896800	4112000
P62857	40S ribosomal protein S28	RPS28	sp|P62857|RS28_HUMAN 40S ribosomal protein S2	2	7.8409	3.7217	3.2677	1.1842	3.2677	18987000	32783000	6557100
P62873;F6U	Guanine nucleotide-binding protein G(I)/G(	GNB1	sp|P62873|GBB1_HUMAN Guanine nucleotide-bin	13	37.377	3.2364	4.2746	1.6165	3.2364	2812200	17175000	35389000
Q7L7X3;J3Q	Serine/threonine-protein kinase TAO1	TAOK1	sp|Q7L7X3|TAOK1_HUMAN Serine/threonine-prot	15	116.07	1.3856	3.2072	3.0804	3.0804	2889600	6633200	6110200
O75955;A0A	Flotillin-1	FLOT1	sp|O75955|FLOT1_HUMAN Flotillin-1 OS=Homo sa	16	47.355	3.0802	3.6951	1.7534	3.0802	11366000	21730000	4780700
Q12899;A2A	Tripartite motif-containing protein 26	TRIM26	sp|Q12899|TRI26_HUMAN Tripartite motif-contain	7	62.165	4.126		2.001	3.0635	1519900	2525100	1427900
Q99986;H0Y	Serine/threonine-protein kinase VRK1	VRK1	sp|Q99986|VRK1_HUMAN Serine/threonine-prote	14	45.476	3.4612	3.0568	1.1048	3.0568	2738700	26700000	18471000
C9J2Y9;P30	DNA-directed RNA polymerase;DNA-direct	POLR2B	tr|C9J2Y9|C9J2Y9_HUMAN DNA-directed RNA poly	34	133.06	3.7497	3.0177	3.0513	3.0513	4282800	9848300	392790
H3BV80;H3	RNA-binding protein with serine-rich doma	RNPS1	tr|H3BV80|H3BV80_HUMAN RNA-binding protein	6	24.561	3.0441	3.3076	2.5543	3.0441	139510000	204410000	94293000
Q9UBI6	Guanine nucleotide-binding protein G(I)/G(	GNG12	sp|Q9UBI6|GBG12_HUMAN Guanine nucleotide-bi	3	8.0061	3.0438	4.9367	2.0117	3.0438	3890300	34430000	84342000

Continued on next page

<table>
<tr><th>Protein ID</th><th>Protein name</th><th>Gene name</th><th>Fasta headers</th><th>Unique peptides</th><th>Mol. weight [kDa]</th><th>Norm. ratio [H/L] Rep.1</th><th>Norm. ratio [H/L] Rep.2</th><th>Norm. ratio [H/L] Rep.3</th><th>Median norm. ratio</th><th>iBAQ [H] Exp.1</th><th>iBAQ [H] Exp.2</th><th>iBAQ [H] Exp.3</th></tr>
<tr><td>I1E4Y6;Q6Y7</td><td>PERQ amino acid-rich with GYF domain-cont</td><td>GIGYF2</td><td>tr|I1E4Y6|I1E4Y6_HUMAN GRB10-interacting GYF pı</td><td>11</td><td>152.53</td><td>3.4506</td><td>2.6293</td><td></td><td>3.03995</td><td>571610</td><td>1017200</td><td>0</td></tr>
<tr><td>P16403;P10</td><td>Histone H1.2;Histone H1.4</td><td>HIST1H1C;HIS</td><td>sp|P16403|H12_HUMAN Histone H1.2 OS=Homo sa</td><td>12</td><td>21.364</td><td>3.0264</td><td>2.2983</td><td>3.593</td><td>3.0264</td><td>103310000</td><td>303900000</td><td>129810000</td></tr>
<tr><td>P19387;H3B</td><td>DNA-directed RNA polymerase II subunit RF</td><td>POLR2C</td><td>sp|P19387|RPB3_HUMAN DNA-directed RNA polyr</td><td>10</td><td>31.441</td><td>3.0233</td><td>3.8035</td><td>2.6379</td><td>3.0233</td><td>11207000</td><td>18173000</td><td>2879500</td></tr>
<tr><td>Q5HYB6</td><td></td><td>DKFZp686J13</td><td>tr|Q5HYB6|Q5HYB6_HUMAN Epididymis luminal pr</td><td>21</td><td>27.175</td><td>4.5555</td><td>2.9635</td><td>2.9779</td><td>2.9779</td><td>1471400</td><td>6297900</td><td>1443000</td></tr>
<tr><td>P06748;E5R</td><td>Nucleophosmin</td><td>NPM1</td><td>sp|P06748|NPM_HUMAN Nucleophosmin OS=Hom</td><td>17</td><td>32.575</td><td>3.5369</td><td>2.9192</td><td>1.7384</td><td>2.9192</td><td>45741000</td><td>124660000</td><td>102380000</td></tr>
<tr><td>J3QLD9;E7E</td><td>Flotillin-2</td><td>FLOT2</td><td>tr|J3QLD9|J3QLD9_HUMAN Flotillin-2 OS=Homo sa</td><td>15</td><td>47.142</td><td>2.9067</td><td>4.1686</td><td>1.6969</td><td>2.9067</td><td>8440000</td><td>26271000</td><td>11510000</td></tr>
<tr><td>P62879;C9JI</td><td>Guanine nucleotide-binding protein G(I)/G(</td><td>GNB2</td><td>sp|P62879|GBB2_HUMAN Guanine nucleotide-binc</td><td>12</td><td>37.331</td><td>2.9058</td><td>4.836</td><td>1.5201</td><td>2.9058</td><td>4881400</td><td>14551000</td><td>15388000</td></tr>
<tr><td>Q53H12;E9F</td><td>Acylglycerol kinase, mitochondrial</td><td>AGK</td><td>sp|Q53H12|AGK_HUMAN Acylglycerol kinase, mitc</td><td>18</td><td>47.137</td><td>1.1258</td><td>2.8627</td><td>4.0535</td><td>2.8627</td><td>4820100</td><td>15152000</td><td>13972000</td></tr>
<tr><td>Q13112</td><td>Chromatin assembly factor 1 subunit B</td><td>CHAF1B</td><td>sp|Q13112|CAF1B_HUMAN Chromatin assembly fa</td><td>4</td><td>61.492</td><td>2.844</td><td>2.8809</td><td></td><td>2.86245</td><td>512590</td><td>77757</td><td>0</td></tr>
<tr><td>A0A087WV.</td><td>DNA-directed RNA polymerases I, II, and III</td><td>POLR2E</td><td>tr|A0A087WVZ9|A0A087WVZ9_HUMAN DNA-direc</td><td>7</td><td>21.459</td><td>3.1167</td><td>2.4935</td><td></td><td>2.8051</td><td>3322300</td><td>15849000</td><td>0</td></tr>
<tr><td>Q92820</td><td>Gamma-glutamyl hydrolase</td><td>GGH</td><td>sp|Q92820|GGH_HUMAN Gamma-glutamyl hydrola</td><td>8</td><td>35.964</td><td>2.8025</td><td>5.1253</td><td>2.0979</td><td>2.8025</td><td>2133200</td><td>7381800</td><td>1489700</td></tr>
<tr><td>Q96GA3;A0</td><td>Protein LTV1 homolog</td><td>LTV1</td><td>sp|Q96GA3|LTV1_HUMAN Protein LTV1 homolog C</td><td>15</td><td>54.854</td><td>1.3874</td><td>3.0356</td><td>2.7805</td><td>2.7805</td><td>11848000</td><td>15414000</td><td>11793000</td></tr>
<tr><td>P60709;A0A</td><td>Actin, cytoplasmic 1;Actin, cytoplasmic 1, N-</td><td>ACTB</td><td>sp|P60709|ACTB_HUMAN Actin, cytoplasmic 1 OS=</td><td>27</td><td>41.736</td><td>1.2178</td><td>3.5005</td><td>2.7751</td><td>2.7751</td><td>11323000</td><td>36019000</td><td>20782000</td></tr>
<tr><td>Added01;CON__Q9U6Y5;I3L170</td><td></td><td></td><td>tr|Added01|TauYfp_HUMAN Tau Yfp</td><td>39</td><td>43.256</td><td>3.5063</td><td>2.7256</td><td>2.1606</td><td>2.7256</td><td>4130100000</td><td>8914000000</td><td>3833400000</td></tr>
<tr><td>Q99613;B5N</td><td>Eukaryotic translation initiation factor 3 sub</td><td>EIF3C;EIF3CL</td><td>sp|Q99613|EIF3C_HUMAN Eukaryotic translation ir</td><td>33</td><td>105.34</td><td>2.5577</td><td>2.8357</td><td></td><td>2.6967</td><td>855670</td><td>4670000</td><td>443520</td></tr>
<tr><td>P28066</td><td>Proteasome subunit alpha type-5</td><td>PSMA5</td><td>sp|P28066|PSA5_HUMAN Proteasome subunit alpl</td><td>13</td><td>26.411</td><td></td><td>3.108</td><td>2.254</td><td>2.681</td><td>713200</td><td>3627100</td><td>526020</td></tr>
<tr><td>Q9HCM4</td><td>Band 4.1-like protein 5</td><td>EPB41L5</td><td>sp|Q9HCM4|E41L5_HUMAN Band 4.1-like protein 5</td><td>9</td><td>81.855</td><td>2.1785</td><td>3.0182</td><td></td><td>2.59835</td><td>227100</td><td>3722200</td><td>0</td></tr>
<tr><td>A0A087WY\</td><td>Slit homolog 2 protein;Slit homolog 2 prote</td><td>SLIT2</td><td>tr|A0A087WYV5|A0A087WYV5_HUMAN Slit homol</td><td>9</td><td>159.98</td><td>2.0219</td><td>3.1071</td><td></td><td>2.5645</td><td>487410</td><td>1400300</td><td>599630</td></tr>
<tr><td>A0A1W2PQ</td><td>DNA repair protein RAD50</td><td>RAD50</td><td>tr|A0A1W2PQ90|A0A1W2PQ90_HUMAN Uncharact</td><td>25</td><td>142.94</td><td>2.5131</td><td>3.214</td><td>0.89296</td><td>2.5131</td><td>1048700</td><td>5250600</td><td>1514700</td></tr>
<tr><td>Q9H0U4;E9I</td><td>Ras-related protein Rab-1B;Putative Ras-rel</td><td>RAB1B;RAB1(</td><td>sp|Q9H0U4|RAB1B_HUMAN Ras-related protein Ra</td><td>12</td><td>22.171</td><td></td><td>2.7446</td><td>2.1603</td><td>2.45245</td><td>682330</td><td>3902100</td><td>564680</td></tr>
<tr><td>Q13823;H0Y</td><td>Nucleolar GTP-binding protein 2</td><td>GNL2</td><td>sp|Q13823|NOG2_HUMAN Nucleolar GTP-binding</td><td>18</td><td>83.654</td><td>2.5228</td><td>2.3703</td><td></td><td>2.44655</td><td>504360</td><td>3938100</td><td>57885</td></tr>
<tr><td>Q07021;I3L3</td><td>Complement component 1 Q subcomponen</td><td>C1QBP</td><td>sp|Q07021|C1QBP_HUMAN Complement compone</td><td>10</td><td>31.362</td><td>2.1285</td><td>5.1114</td><td>2.4395</td><td>2.4395</td><td>5133600</td><td>39902000</td><td>12700000</td></tr>
<tr><td>Q7Z417</td><td>Nuclear fragile X mental retardation-interac</td><td>NUFIP2</td><td>sp|Q7Z417|NUFP2_HUMAN Nuclear fragile X ment</td><td>15</td><td>76.12</td><td>2.9962</td><td>2.4364</td><td>2.0328</td><td>2.4364</td><td>5829600</td><td>7837800</td><td>5467700</td></tr>
<tr><td>P08670;B0Y</td><td>Vimentin</td><td>VIM</td><td>sp|P08670|VIME_HUMAN Vimentin OS=Homo sapi</td><td>45</td><td>53.651</td><td>2.4027</td><td>2.6728</td><td>1.6894</td><td>2.4027</td><td>59024000</td><td>131930000</td><td>146780000</td></tr>
<tr><td>P19525;C9J</td><td>Interferon-induced, double-stranded RNA-a</td><td>EIF2AK2</td><td>sp|P19525|E2AK2_HUMAN Interferon-induced, do</td><td>12</td><td>62.094</td><td>2.5648</td><td>2.384</td><td>2.0421</td><td>2.384</td><td>145870</td><td>749090</td><td>114650</td></tr>
<tr><td>Q01082</td><td>Spectrin beta chain, non-erythrocytic 1</td><td>SPTBN1</td><td>sp|Q01082|SPTB2_HUMAN Spectrin beta chain, no</td><td>120</td><td>274.61</td><td>2.354</td><td>5.0002</td><td>1.5658</td><td>2.354</td><td>14363000</td><td>46226000</td><td>27935000</td></tr>
<tr><td>A0A0D9SF54;A0A0D9SGF6;A0A0D9SFF6;A0A0D9SFH4;A0</td><td></td><td>SPTAN1</td><td>tr|A0A0D9SF54|A0A0D9SF54_HUMAN Spectrin alpl</td><td>132</td><td>282.83</td><td>2.3502</td><td>5.0256</td><td>1.4746</td><td>2.3502</td><td>16833000</td><td>62862000</td><td>26440000</td></tr>
<tr><td>P07948;E5R</td><td>Tyrosine-protein kinase Lyn</td><td>LYN</td><td>sp|P07948|LYN_HUMAN Tyrosine-protein kinase Ly</td><td>12</td><td>58.573</td><td></td><td>2.3857</td><td>2.2951</td><td>2.3404</td><td>0</td><td>1032300</td><td>2217900</td></tr>
<tr><td>Q9UN86;D6</td><td>Ras GTPase-activating protein-binding prote</td><td>G3BP2</td><td>sp|Q9UN86|G3BP2_HUMAN Ras GTPase-activating</td><td>11</td><td>54.12</td><td>2.3338</td><td>1.3159</td><td>2.5495</td><td>2.3338</td><td>8272900</td><td>13070000</td><td>6033900</td></tr>
<tr><td>P07900;G3V</td><td>Heat shock protein HSP 90-alpha</td><td>HSP90AA1</td><td>sp|P07900|HS90A_HUMAN Heat shock protein HSP</td><td>63</td><td>84.659</td><td>2.277</td><td>4.0858</td><td>2.008</td><td>2.277</td><td>6729400</td><td>12647000</td><td>4596800</td></tr>
<tr><td>P46013</td><td>Antigen KI-67</td><td>MKI67</td><td>sp|P46013|KI67_HUMAN Proliferation marker prot</td><td>17</td><td>358.69</td><td>2.3191</td><td>2.2468</td><td>2.2169</td><td>2.2468</td><td>524560</td><td>475360</td><td>44507</td></tr>
<tr><td>P60228;E5R</td><td>Eukaryotic translation initiation factor 3 sub</td><td>EIF3E</td><td>sp|P60228|EIF3E_HUMAN Eukaryotic translation in</td><td>25</td><td>52.22</td><td>2.3804</td><td>2.2127</td><td>1.9171</td><td>2.2127</td><td>1089300</td><td>1573400</td><td>1367200</td></tr>
<tr><td>P49916;K7E</td><td>DNA ligase 3</td><td>LIG3</td><td>sp|P49916|DNLI3_HUMAN DNA ligase 3 OS=Homo</td><td>8</td><td>112.91</td><td>2.9178</td><td>2.203</td><td>2.1767</td><td>2.203</td><td>1532800</td><td>1039000</td><td>946440</td></tr>
<tr><td>Q5RKV6</td><td>Exosome complex component MTR3</td><td>EXOSC6</td><td>sp|Q5RKV6|EXOS6_HUMAN Exosome complex con</td><td>6</td><td>28.235</td><td>2.3573</td><td>2.1818</td><td>2.111</td><td>2.1818</td><td>11071000</td><td>15292000</td><td>4620400</td></tr>
<tr><td>P12931</td><td>Proto-oncogene tyrosine-protein kinase Src</td><td>SRC</td><td>sp|P12931|SRC_HUMAN Proto-oncogene tyrosine-</td><td>8</td><td>59.834</td><td>2.168</td><td>4.1415</td><td>1.8886</td><td>2.168</td><td>748320</td><td>2601200</td><td>1702500</td></tr>
<tr><td>P63092;Q5J</td><td>Guanine nucleotide-binding protein G(s) su</td><td>GNAS</td><td>sp|P63092|GNAS2_HUMAN Guanine nucleotide-bi</td><td>13</td><td>45.664</td><td>2.1627</td><td>4.1417</td><td>1.5402</td><td>2.1627</td><td>1488700</td><td>9048500</td><td>3740200</td></tr>
<tr><td>O75531;E9P</td><td>Barrier-to-autointegration factor;Barrier-to-</td><td>BANF1</td><td>sp|O75531|BAF_HUMAN Barrier-to-autointegratio</td><td>6</td><td>10.058</td><td>2.1605</td><td>2.4895</td><td>1.4442</td><td>2.1605</td><td>167700000</td><td>464450000</td><td>175040000</td></tr>
<tr><td>Q9Y265;E7E</td><td>RuvB-like 1</td><td>RUVBL1</td><td>sp|Q9Y265|RUVB1_HUMAN RuvB-like 1 OS=Homo</td><td>24</td><td>50.227</td><td>1.8486</td><td>2.1597</td><td>2.5745</td><td>2.1597</td><td>2800700</td><td>6342500</td><td>382330</td></tr>
<tr><td>O14578;H7E</td><td>Citron Rho-interacting kinase</td><td>CIT</td><td>sp|O14578|CTRO_HUMAN Citron Rho-interacting k</td><td>11</td><td>231.43</td><td>3.1776</td><td>2.1397</td><td>0.96079</td><td>2.1397</td><td>57864</td><td>292140</td><td>49921</td></tr>
<tr><td>P27986;H0Y</td><td>Phosphatidylinositol 3-kinase regulatory su</td><td>PIK3R1</td><td>sp|P27986|P85A_HUMAN Phosphatidylinositol 3-k</td><td>6</td><td>83.597</td><td>1.5657</td><td>2.1169</td><td>2.5085</td><td>2.1169</td><td>745890</td><td>1116000</td><td>1210500</td></tr>
<tr><td>Q6WCQ1;J3</td><td>Myosin phosphatase Rho-interacting protei</td><td>MPRIP</td><td>sp|Q6WCQ1|MPRIP_HUMAN Myosin phosphatase</td><td>12</td><td>116.53</td><td>2.1049</td><td>2.173</td><td>0.79734</td><td>2.1049</td><td>588250</td><td>1624600</td><td>104110</td></tr>
<tr><td>P08754</td><td>Guanine nucleotide-binding protein G(k) su</td><td>GNAI3</td><td>sp|P08754|GNAI3_HUMAN Guanine nucleotide-bir</td><td>15</td><td>40.532</td><td>2.0157</td><td>4.9059</td><td>2.0813</td><td>2.0813</td><td>19273000</td><td>75469000</td><td>42160000</td></tr>
<tr><td>O15234;J3K</td><td>Protein CASC3</td><td>CASC3</td><td>sp|O15234|CASC3_HUMAN Protein CASC3 OS=Hom</td><td>8</td><td>76.277</td><td>2.067</td><td>2.0138</td><td>2.7423</td><td>2.067</td><td>698630</td><td>2073400</td><td>4126900</td></tr>
<tr><td>P17987;E7E</td><td>T-complex protein 1 subunit alpha</td><td>TCP1</td><td>sp|P17987|TCPA_HUMAN T-complex protein 1 sub</td><td>34</td><td>60.343</td><td>1.5294</td><td>2.2873</td><td>2.0582</td><td>2.0582</td><td>485300</td><td>1703400</td><td>621710</td></tr>
<tr><td>Q5SRQ6;P6</td><td>Casein kinase II subunit beta</td><td>CSNK2B;CSN</td><td>tr|Q5SRQ6|Q5SRQ6_HUMAN Casein kinase II subui</td><td>8</td><td>26.925</td><td>2.0436</td><td>2.4349</td><td>1.2233</td><td>2.0436</td><td>5899500</td><td>15187000</td><td>987590</td></tr>
<tr><td>P08238;Q58</td><td>Heat shock protein HSP 90-beta</td><td>HSP90AB1</td><td>sp|P08238|HS90B_HUMAN Heat shock protein HSP</td><td>64</td><td>83.263</td><td>1.9891</td><td>2.9312</td><td>2.02</td><td>2.02</td><td>7615400</td><td>29148000</td><td>15600000</td></tr>
<tr><td>O95425;A0A</td><td>Supervillin</td><td>SVIL</td><td>sp|O95425|SVIL_HUMAN Supervillin OS=Homo sap</td><td>9</td><td>247.74</td><td>1.4281</td><td>3.0818</td><td>2.0134</td><td>2.0134</td><td>360680</td><td>1636100</td><td>213390</td></tr>
<tr><td>Q14676;A2A</td><td>Mediator of DNA damage checkpoint protei</td><td>MDC1</td><td>sp|Q14676|MDC1_HUMAN Mediator of DNA damag</td><td>20</td><td>226.66</td><td>2.1653</td><td>2.0075</td><td>1.5305</td><td>2.0075</td><td>303620</td><td>152960</td><td>60101</td></tr>
<tr><td>Q14008;H0Y</td><td>Cytoskeleton-associated protein 5</td><td>CKAP5</td><td>sp|Q14008|CKAP5_HUMAN Cytoskeleton-associate</td><td>72</td><td>225.49</td><td>1.7242</td><td>2.0021</td><td>2.3599</td><td>2.0021</td><td>325010</td><td>2252600</td><td>1461800</td></tr>
</table>

Uncropped scans of blots

Supplementary Fig. 1c

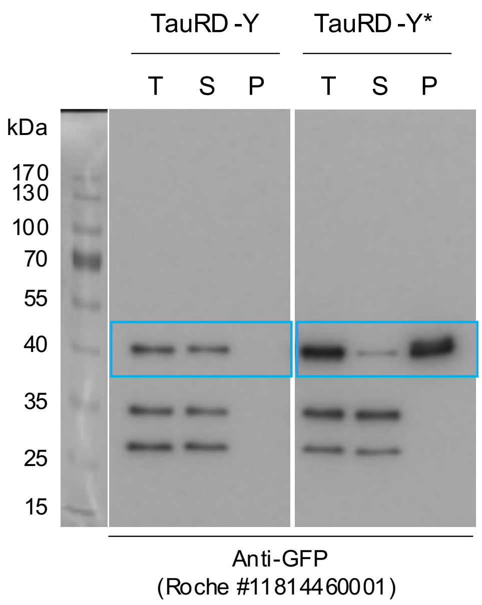

Supplementary Fig. 1e

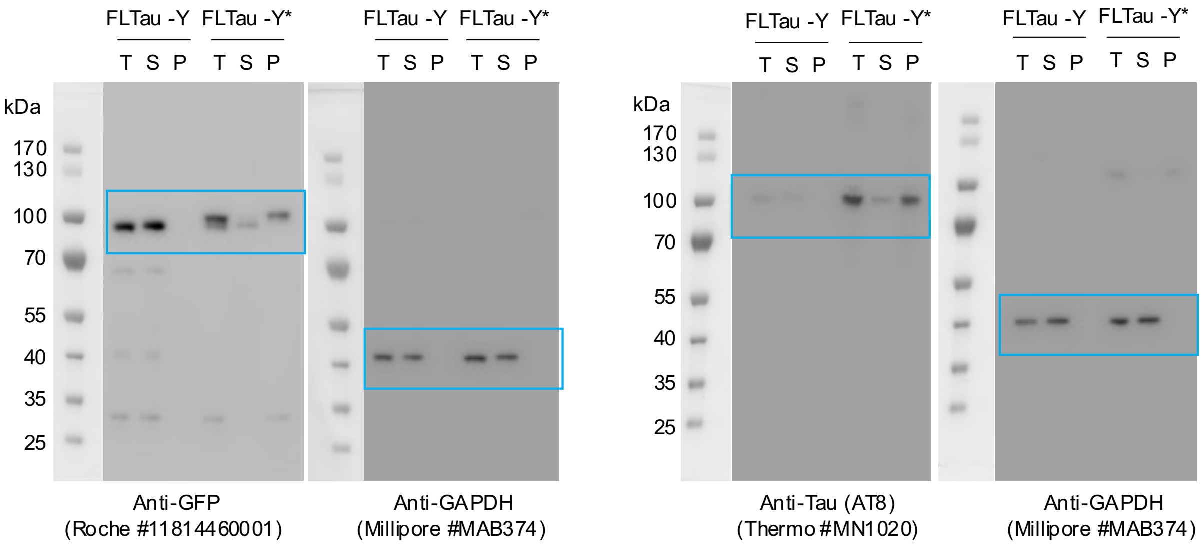

Supplementary Fig. 3a

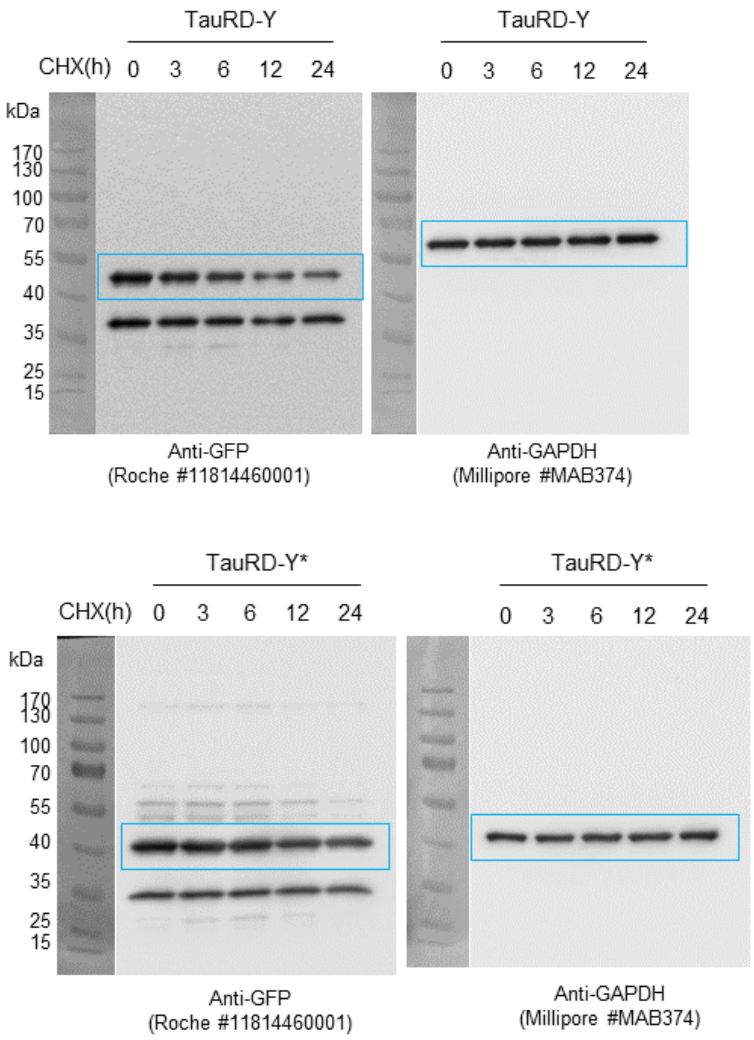

Supplementary Fig. 3b

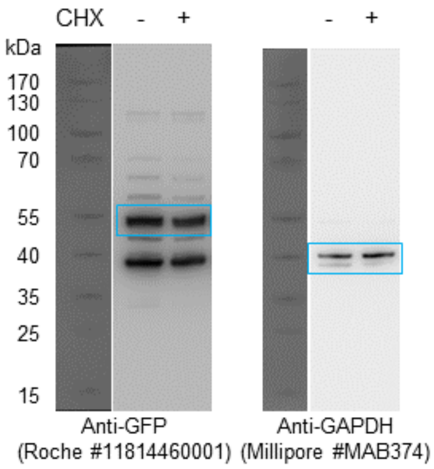

Supplementary Fig. 3c

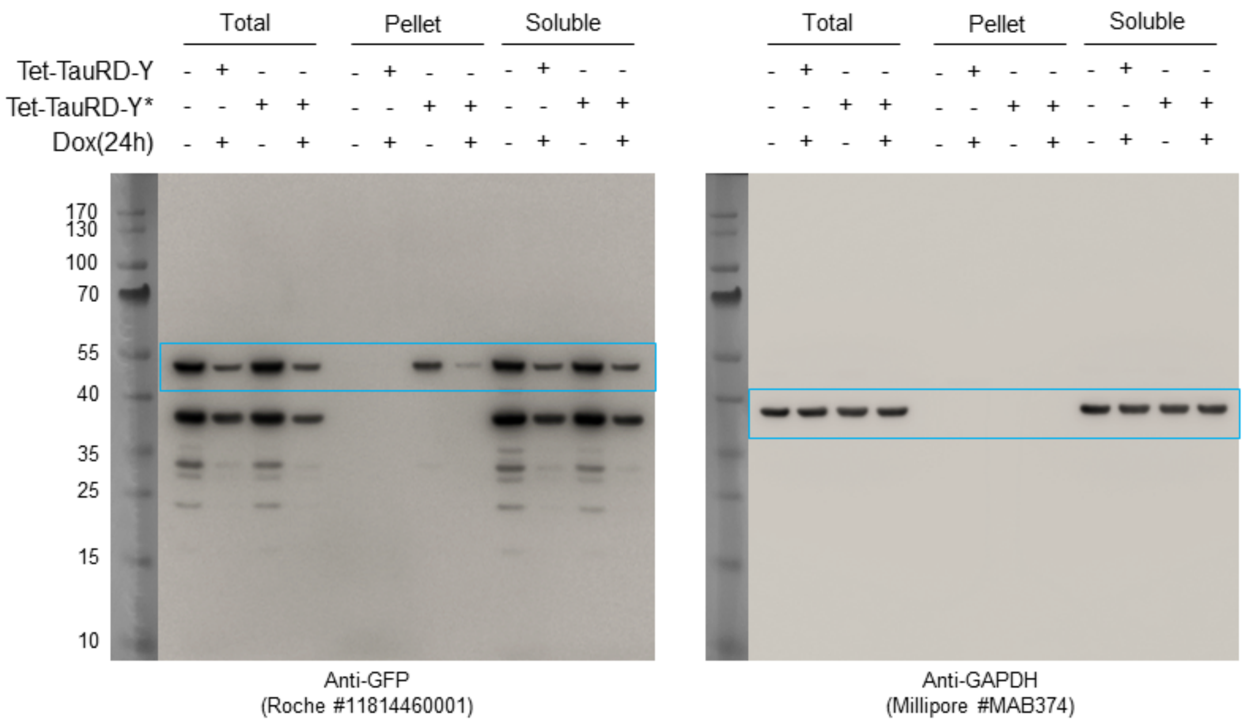

Supplementary Fig. 3c

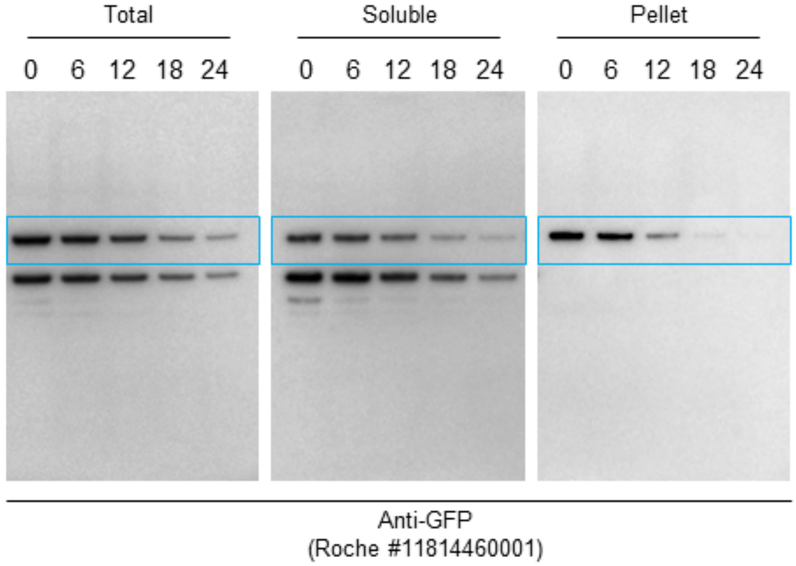

Supplementary Fig. 4a

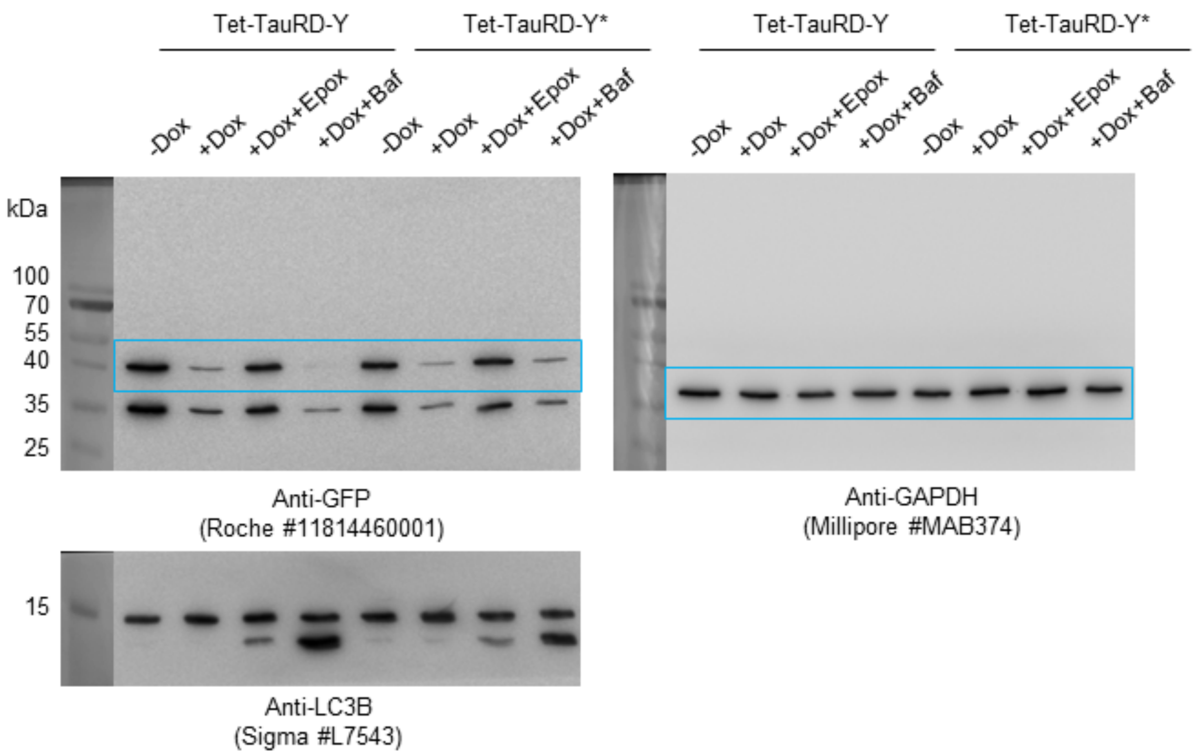

Supplementary Fig. 4b

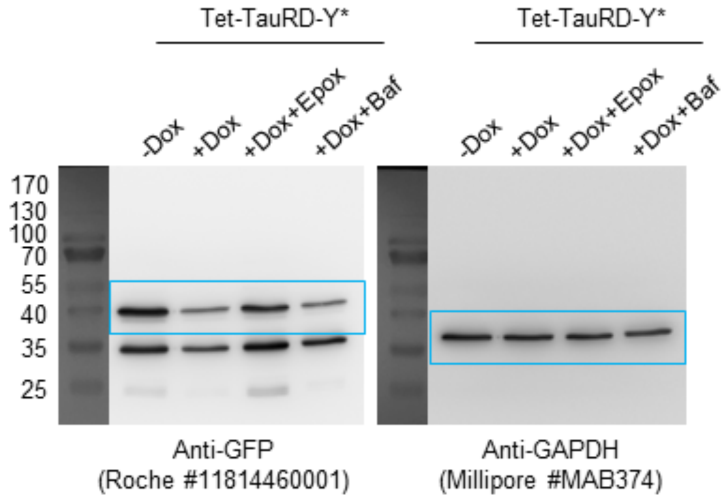

Supplementary Fig. 4e

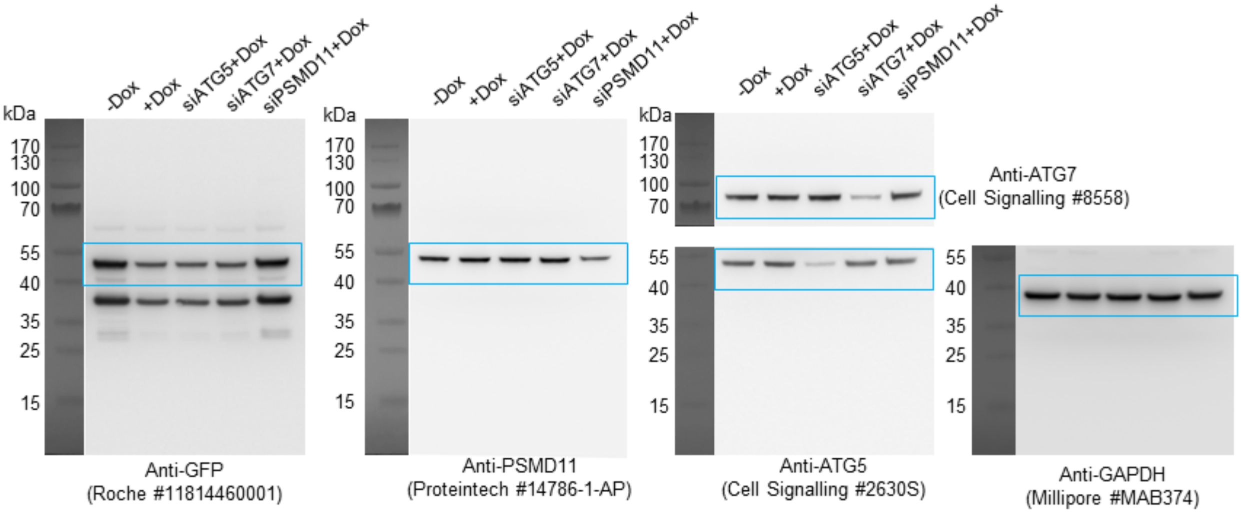

Supplementary Fig. 5e

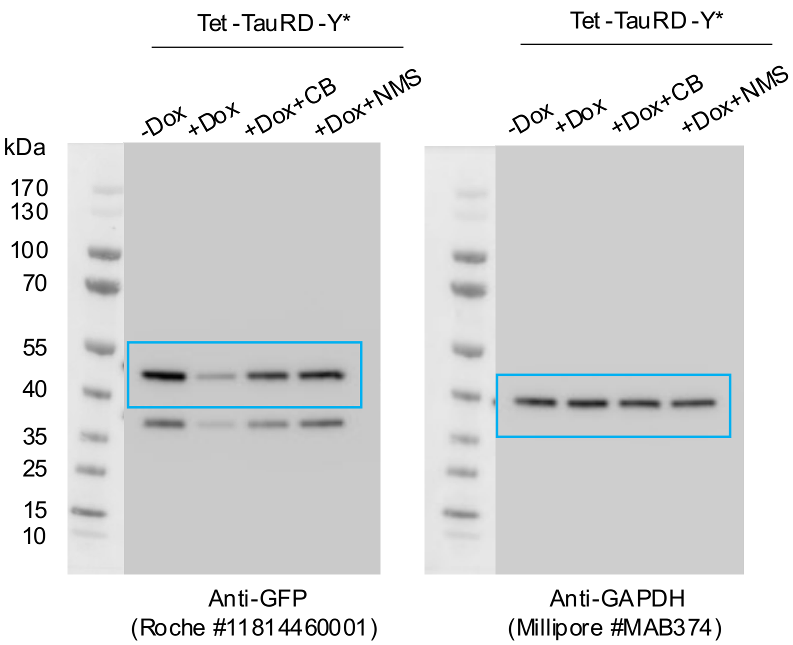

Supplementary Fig. 5g

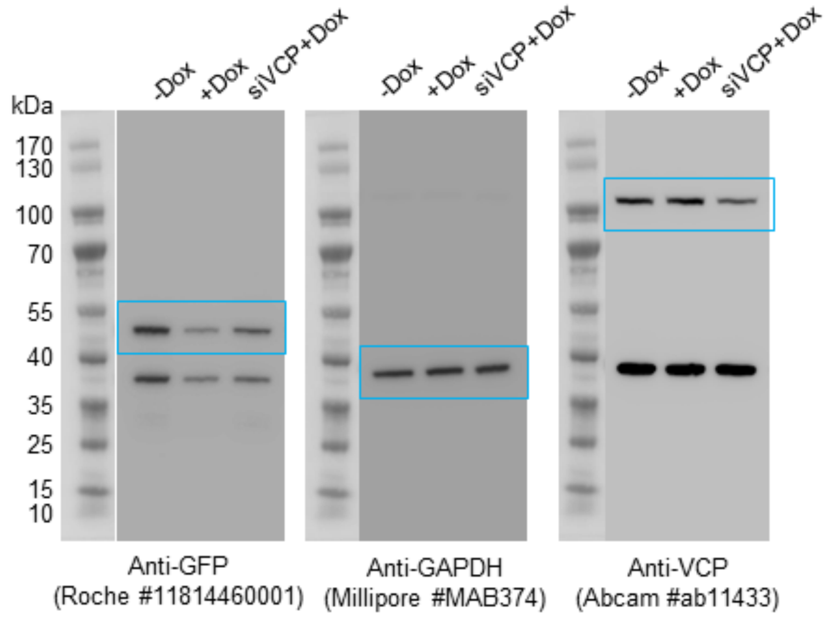

Supplementary Fig. 5i

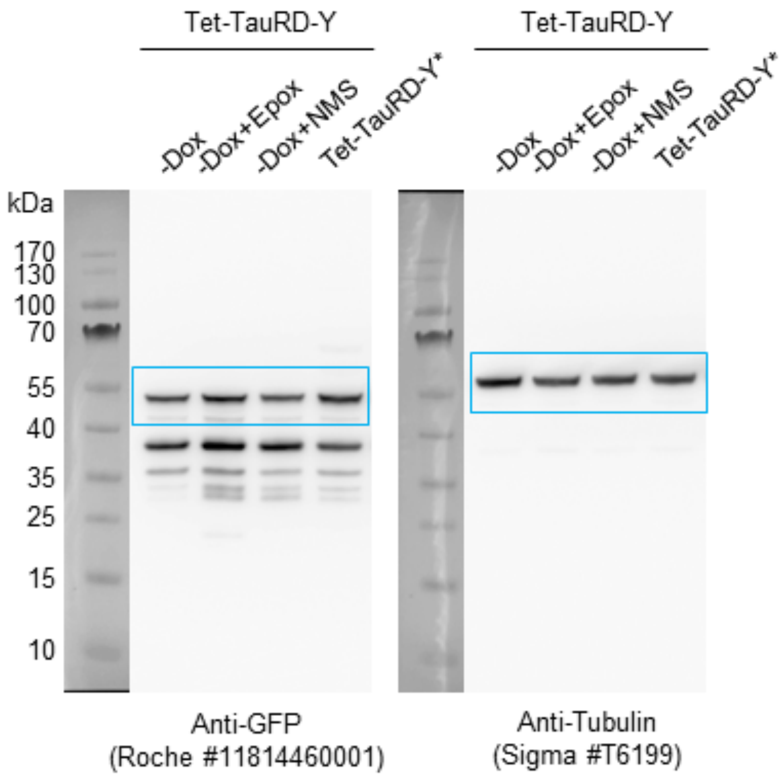

Supplementary Fig. 5k

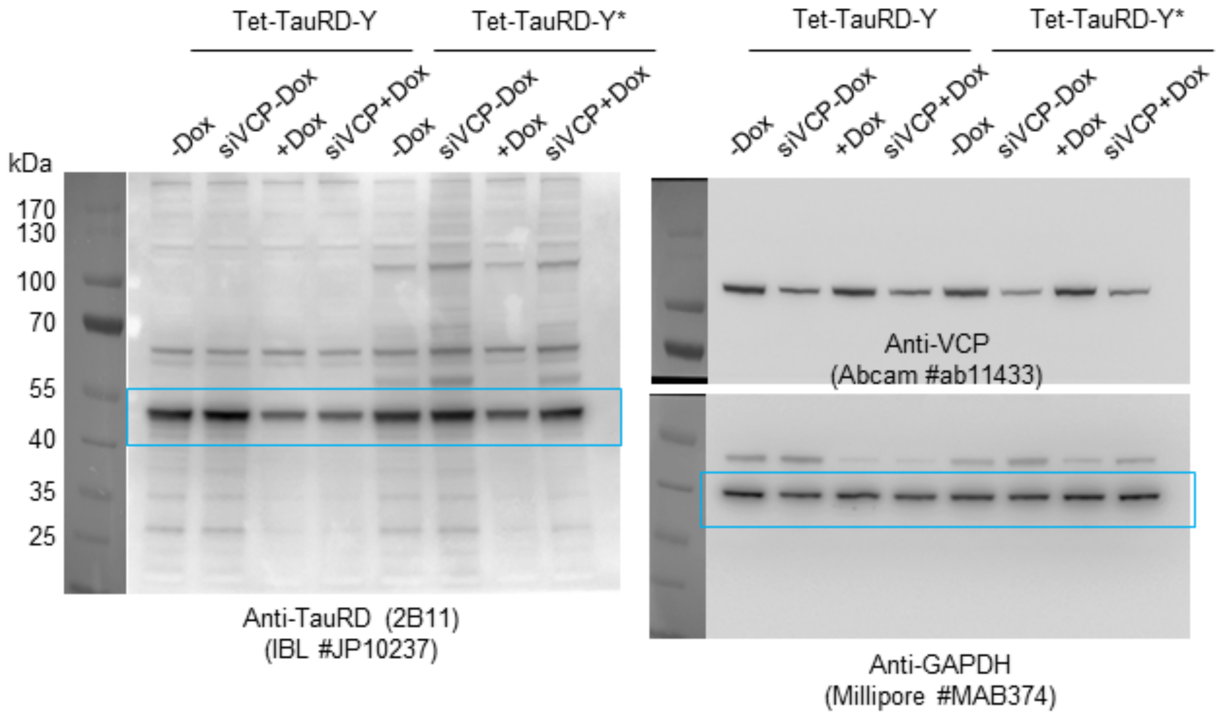

Supplementary Fig. 6c

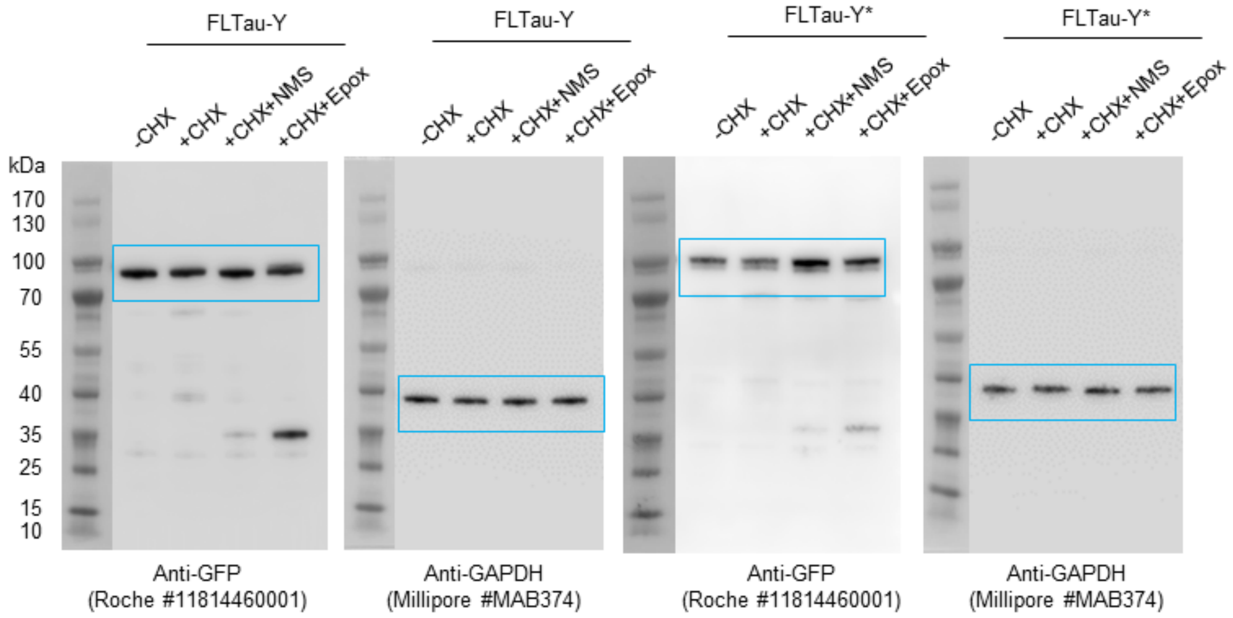

Supplementary Fig. 6e

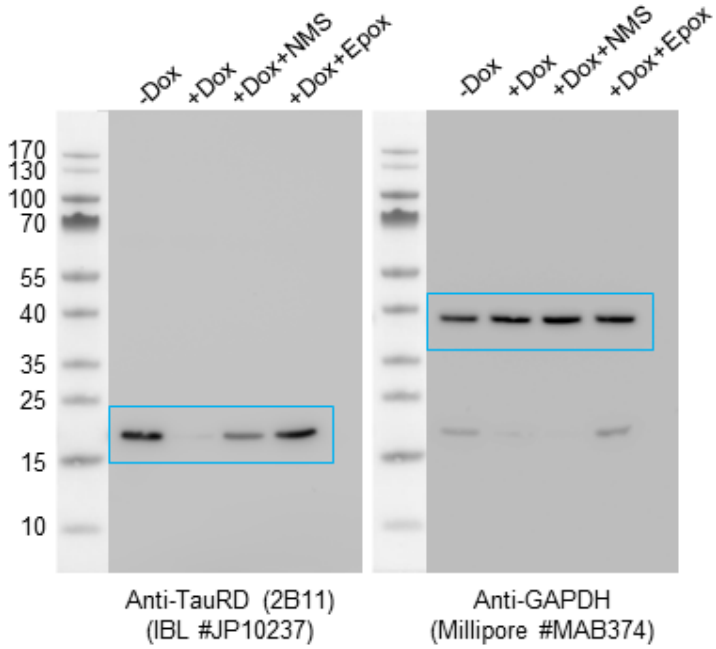

Supplementary Fig. 8e

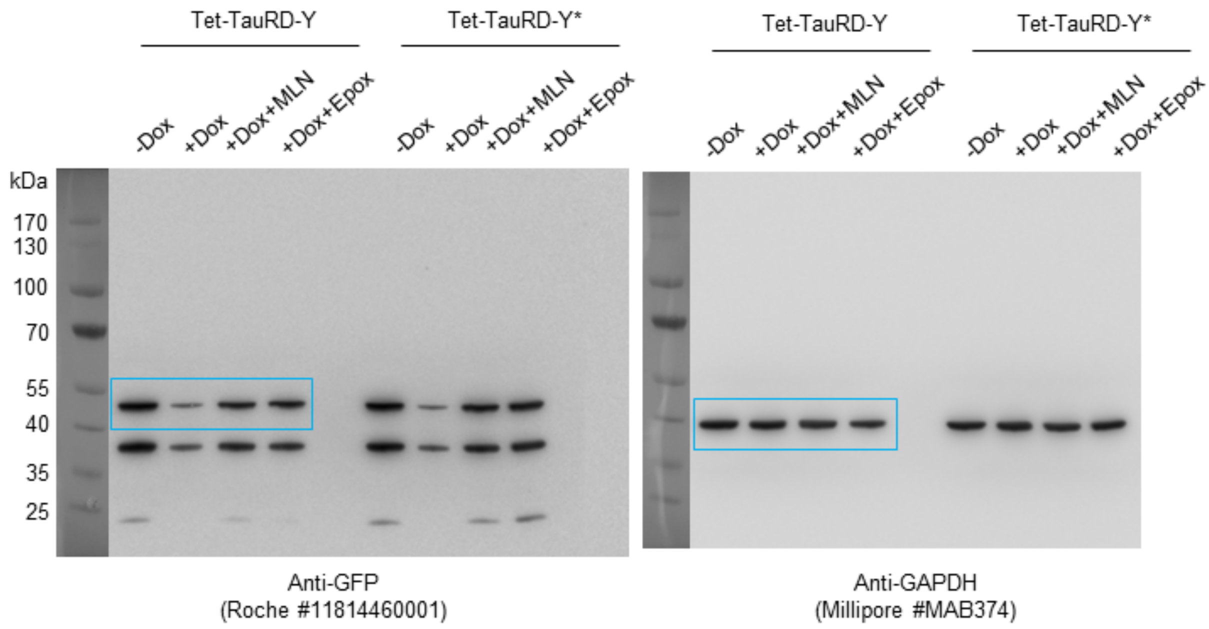

Supplementary Fig. 12c

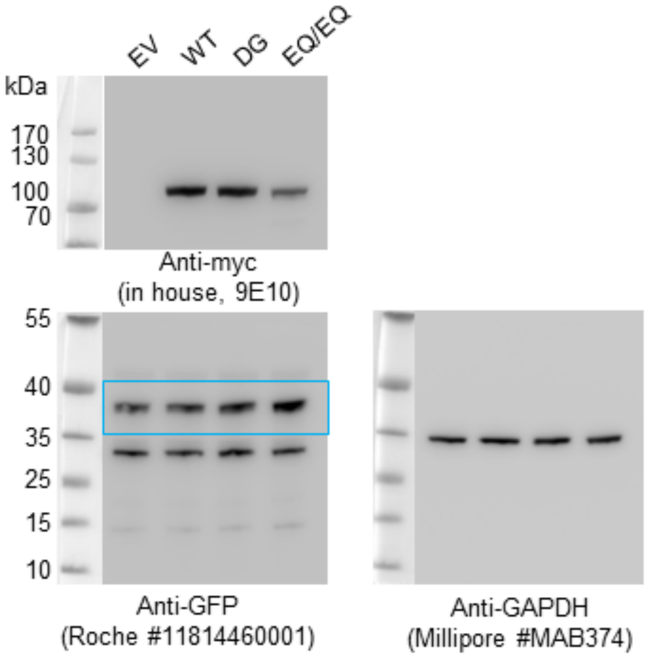

277
