# Supplementary material for: The AAA+ chaperone VCP disaggregates Tau fibrils and generates aggregate seeds in a cellular system
Source: Nat Commun. 2023 Feb 2;14:560. doi: 10.1038/s41467-023-36058-2 (PMC9894937; doi:10.1038/s41467-023-36058-2)
Supplement: Supplementary file 3 — Reporting Summary [file 41467_2023_36058_MOESM3_ESM.pdf]

nature portfolio

Corresponding author(s): Mark S. Hipp and F. Ulrich Hartl

Last updated by author(s): Jan 13, 2023

Reporting Summary

Nature Portfolio wishes to improve the reproducibility of the work that we publish. This form provides structure for consistency and transparency in reporting. For further information on Nature Portfolio policies, see our Editorial Policies and the Editorial Policy Checklist.

Statistics

For all statistical analyses, confirm that the following items are present in the figure legend, table legend, main text, or Methods section.

n/a	Confirmed	
☐	☒	The exact sample size (*n*) for each experimental group/condition, given as a discrete number and unit of measurement
☐	☒	A statement on whether measurements were taken from distinct samples or whether the same sample was measured repeatedly
☐	☒	The statistical test(s) used AND whether they are one- or two-sided<br>*Only common tests should be described solely by name; describe more complex techniques in the Methods section.*
☒	☐	A description of all covariates tested
☐	☒	A description of any assumptions or corrections, such as tests of normality and adjustment for multiple comparisons
☐	☒	A full description of the statistical parameters including central tendency (e.g. means) or other basic estimates (e.g. regression coefficient) AND variation (e.g. standard deviation) or associated estimates of uncertainty (e.g. confidence intervals)
☐	☒	For null hypothesis testing, the test statistic (e.g. *F*, *t*, *r*) with confidence intervals, effect sizes, degrees of freedom and *P* value noted<br>*Give P values as exact values whenever suitable.*
☒	☐	For Bayesian analysis, information on the choice of priors and Markov chain Monte Carlo settings
☒	☐	For hierarchical and complex designs, identification of the appropriate level for tests and full reporting of outcomes
☒	☐	Estimates of effect sizes (e.g. Cohen's *d*, Pearson's *r*), indicating how they were calculated

Our web collection on statistics for biologists contains articles on many of the points above.

Software and code

Policy information about availability of computer code

Data collection	ZEN 2011 SP7 (black) v14.0.2.201 (Zeiss LSM 780) - Confocal image acquisition<br>LAS X v3.5.7.23225 (Leica SP8 FALCON) - Confocal image acquisition<br>LAS X v3.5.7.23225 (Leica TCS SP8) - Confocal image acquisition<br>LAS-4000 image analyzer (FujiFilm)- Immunoblot acquisition<br>ImageQuant 800 control Software v1.2.0 (Amersham)- Immunoblot acquisition<br>Attune NxT Software v2.4-3.1- Fluorescence-activated cell sorting<br>FACSDiva v6.1.3 - FRET measurement<br>MAPS v2.1 (FEI), K2align, IMOD package v4.9.0, TomoSegMemTV v1.0- Cryo-electron tomography workflow softwares
Data analysis	ImageJ v1.49s - Confocal image analysis and processing<br>Adobe Photoshop CC 2018- Image processing<br>AIDA Image Analyzer v4.27.039- Immunoblot quantification<br>MS Excel 2019- Display of quantitative data and statistics<br>GraphPad Prism 7- Statistics<br>Origin 2019b- Exponential fitting, one-way ANOVA and Tukey post hoc test<br>MaxQuant v1.5.4.1 - Protein identification and SILAC based quantification<br>UNIPROT v2019-03-12- Database for protein identification<br>Perseus v1.2.6.3 - Volcano plot for interactome analysis<br>FlowJo v10.7.1 and V9 - Flow cytometry<br>Amira v6.2 - Cryo-ET<br>SerialEM v3.7.0 - Cryo-ET<br>Motioncor2 v1.2.1 - Cryo-ET

tom_deconv Jun 26, 2019 (https://github.com/dtegunov/tom_deconv) - Cryo-ET
Tomoman Oct 20, 2020 (https://github.com/williamnwan/TOMOMAN) - Cryo-ET

For manuscripts utilizing custom algorithms or software that are central to the research but not yet described in published literature, software must be made available to editors and reviewers. We strongly encourage code deposition in a community repository (e.g. GitHub). See the Nature Portfolio guidelines for submitting code & software for further information.

Data

Policy information about availability of data

All manuscripts must include a data availability statement. This statement should provide the following information, where applicable:

- Accession codes, unique identifiers, or web links for publicly available datasets
- A description of any restrictions on data availability
- For clinical datasets or third party data, please ensure that the statement adheres to our policy

All data supporting the findings of this study are included in the manuscript and the Supplemental Information. Source data are provided with this paper. Additional data is available from the corresponding authors upon reasonable request. The mass spectrometry proteomics data associated to Fig. 2a have been deposited to the ProteomeXchange Consortium via the PRIDE93 partner repository (https://www.ebi.ac.uk/pride/archive/) with the dataset identifier PXD023400. This PRIDE entry additionally contains analyses that are not a part of this study. The tomograms shown in Fig. 1d and Fig. 3c are available in the EMDB (https://www.ebi.ac.uk/emdb/) through the following information: EMD ID: EMD-13739 (Fig. 1d) and EMD-13740 (Fig. 3c).

Field-specific reporting

Please select the one below that is the best fit for your research. If you are not sure, read the appropriate sections before making your selection.

☒ Life sciences ☐ Behavioural & social sciences ☐ Ecological, evolutionary & environmental sciences

For a reference copy of the document with all sections, see nature.com/documents/nr-reporting-summary-flat.pdf

Life sciences study design

All studies must disclose on these points even when the disclosure is negative.

Sample size	No statistical methods were used to predetermine sample size. Based upon accepted standards in the field, quantitative experiments were performed in at least three independent replicates and statistical significance was evaluated. Animal experiment data is only qualitative and obtained from three animals per group.
Data exclusions	The original proteomics experiment shown in Fig. 2a was performed with three SILAC labels- Light (TauRD-Y), Heavy (TauRD-Y*), and Medium (a third cell line that is not a part of this study). The proteomics raw data in the PRIDE entry PXD023400 contains analyses of all the labels. Since the Medium labeled cell line is not a part of this study, the Medium label data was excluded from analysis shown in this study. No data other data were excluded from the rest of the experiments presented in this manuscript.
Replication	Experiments were carried out at least twice or in several independent replicates using protocols outlined in the Methods section. Similar results were observed.
Randomization	Mouse embryos of both sexes were chosen randomly for neuronal cell cultures. Animals of either sex were used in all experimental groups.
Blinding	Investigators were not blinded during the experiments. Blinding was not relevant for this study as no allocation of human/animal subjects was involved.

Reporting for specific materials, systems and methods

We require information from authors about some types of materials, experimental systems and methods used in many studies. Here, indicate whether each material, system or method listed is relevant to your study. If you are not sure if a list item applies to your research, read the appropriate section before selecting a response.

Materials & experimental systems

n/a	Involved in the study	
☐	☒	Antibodies
☐	☒	Eukaryotic cell lines
☒	☐	Palaeontology and archaeology
☐	☒	Animals and other organisms
☒	☐	Human research participants
☒	☐	Clinical data
☒	☐	Dual use research of concern

Methods

n/a	Involved in the study	
☒	☐	ChIP-seq
☐	☒	Flow cytometry
☒	☐	MRI-based neuroimaging

Antibodies

Antibodies used	VCP (AbCam #ab11433), anti-VCP (Novus Biologicals #NB100-1558), anti-GFP (Roche #11814460001), anti-Ubiquitin Lys48-specific (Millipore #05-1307), anti-ubiquitin Lys63-specific (AbCam #ab179434), anti-Ubiquitin (P4D1) (SantaCruz #sc-8017), anti-Tau (pS356) (GeneTex #GTX50165), anti-Phospho-Tau (S202, T205) (Thermo Fisher Scientific # MN1020), anti-NPLOC4 (Sigma #HPA021560), anti-UFD1L (AbCam #ab96648), anti-Ubiquitin FK2 (Millipore #04-263), anti-Tau (Tau-5) (Thermo #MA5-12808), anti-Human Tau/Repeat Domain (2B11) (IBL #JP10237), anti-LC3B (Sigma #L7543), anti-Atg5 (Cell Signalling #2630S), anti-Atg7 (Cell Signalling #8558), anti-PSMD11 (Proteintech #14786-1-AP), anti-GAPDH (Millipore #MAB374), anti-Tubulin (Sigma #T6199). Cy5-conjugated anti-mouse (Thermo #A10524), Cy-5 conjugated anti-rabbit (Thermo # A10523), Alexa Fluor 647 AffiniPure anti-mouse (Jackson ImmunoResearch #715-605-151), anti-mouse IgG peroxidase conjugate (Sigma #A4416), anti-rabbit peroxidase conjugate (Sigma #A9169), IRDye 680RD anti-mouse (LI-COR #926-68070), IRDye 800CW anti-rabbit (LI-COR #926-32211).
Validation	Most validations were performed by manufacturers: VCP (AbCam #ab11433) Abpromise guaranteed Immunocytochemistry (ICC) and western blotting (WB), anti-VCP (Novus Biologicals #NB100-1558) knockdown validated in-house for ICC/WB, anti-GFP (Roche #11814460001) validated for WB, anti-Ubiquitin Lys48-specific (Millipore #05-1307) validated for ICC, anti-ubiquitin Lys63-specific (AbCam #ab179434) validated for immunohistochemistry, anti-Ubiquitin (P4D1) (SantaCruz #sc-8017) validated for WB, anti-Tau (pS356) (GeneTex #GTX50165) validated for ICC, anti-Phospho-Tau (S202, T205) (Thermo Fisher Scientific # MN1020) validated for ICC and WB, anti-NPLOC4 (Sigma #HPA021560) validated for ICC, anti-UFD1L (AbCam #ab96648) validated for ICC, anti-Ubiquitin FK2 (Millipore #04-263) validated for ICC and WB, anti-Tau (Tau-5) (Thermo #MA5-12808) validated for ICC, anti-Human Tau/Repeat Domain (2B11) (IBL #JP10237) validation performed in Yuste-Checa et al., Nat Comm, 2021, anti-LC3B (Sigma #L7543) validated for WB, anti-Atg5 (Cell Signalling #2630S) validated for WB, anti-Atg7 (Cell Signalling #8558) validated for WB, anti-PSMD11 (Proteintech #14786-1-AP) validated for WB, anti-GAPDH (Millipore #MAB374) validated for WB, anti-Tubulin (Sigma #T6199) validated for WB.

Eukaryotic cell lines

Policy information about cell lines

Cell line source(s)	HEK293T cells were purchased from ATCC. Tet-FLTau and Tet-TauRD cell lines were generated in HEK293T background. Stable propagation of TauRD-Y aggregates was verified by YFP fluorescence microscopy.
Authentication	No authentication was performed.
Mycoplasma contamination	Cell lines were not PCR tested for mycoplasma contamination. No unusual DAPI staining was observed by microscopy.
Commonly misidentified lines (See ICLAC register)	None

Animals and other organisms

Policy information about studies involving animals; ARRIVE guidelines recommended for reporting animal research

Laboratory animals	E15.5 CD-1 wild-type mouse embryos of both sexes were used for cortical neuronal cultures (breeding line MpiCrlIcr:CD-1). rTg4510 mice were obtained by crossing tetO-tauP301L mice (JaxLabs, Stock number 015815) to the CamKIIα-tTA line79 (JaxLabs, Stock number 003010) and maintained on a C57BL/6 genetic background. 4-month-old and 16-month-old animals of either sex were used for the experiments. Littermates were used as controls.<br>Mice were housed in a specific pathogen free facility at 22 ± 1.5 °C, 55 ± 5% humidity, 14-hour light / 10-hour dark cycle.
Wild animals	The study did not involve wild animals.
Field-collected samples	The study did not involve animals collected from the field.
Ethics oversight	All experiments involving mice were performed in accordance with the relevant guidelines and regulations of the Government of Upper Bavaria (Germany).

Note that full information on the approval of the study protocol must also be provided in the manuscript.

Flow Cytometry

Plots

Confirm that:

- [x] The axis labels state the marker and fluorochrome used (e.g. CD4-FITC).
- [x] The axis scales are clearly visible. Include numbers along axes only for bottom left plot of group (a 'group' is an analysis of identical markers).
- [x] All plots are contour plots with outliers or pseudocolor plots.
- [x] A numerical value for number of cells or percentage (with statistics) is provided.

Methodology

Sample preparation	Creation of stable cell lines expressing the Tau repeat domain constructs fused to fluorescence proteins: HEK293T cells were

transfected using Lipofectamine (Thermo). Cells were cultured in in Dulbecco's modified Eagle's medium (DMEM, Biochrom) supplemented with 10 % fetal bovine serum (FBS, GIBCO), 2 mM L-glutamine (GIBCO) and 1,000 µg/ml geneticin for selection. Polyclonal and monoclonal cell lines were generated by fluorescence-activated cell sorting. Upon selection, cells were cultured in medium supplemented with 200 µg/ml geneticin (Thermo) and penicillin/streptomycin (Thermo).

Quantification of FRET positive cells: Cells were harvested with TrypL Express Enzyme (Gibco), washed with PBS once and resuspended in PBS for analysis.

Instrument

Creation of stable cell lines: BD FACS Aria III (BD Biosciences) for cell sorting. FITC-A, DAPI-A lasers for YFP/GFP and mTurquoise2 detection, respectively.
Quantification of FRET positive cells: Attune NxT flow cytometer (Thermo Fisher Scientific) for FRET positive cells quantification. To measure mTurquoise2 and FRET fluorescence signals, cells were excited with 405 nm laser light and fluorescence was determined using 440/50 and 530/30 filters, respectively. To measure the YFP fluorescence signal, cells were excited at 488 nm and emission was recorded using a 530/30 filter.

Software

Creation of stable cell lines: FACSDiva Version 6.1.3.
Quantification of FRET positive cells: Analysis were performed with FlowJo V9.

Cell population abundance

Creation of stable cell lines: All sorted cells showed the corresponding fluorescence protein expression.
Quantification of FRET positive cells: For each sample, 50,000 single cells were collected and analyzed.

Gating strategy

Quantification of FRET positive cells: After gating single cells, an additional gate was introduced to exclude YFP-only cells that show a false-positive signal in the FRET channel due to excitation at 405 nm. The FRET positive gate was set by plotting the FRET fluorescence signal versus the mTurquoise2 fluorescence signal using non-seeded cells as reference.

☒ Tick this box to confirm that a figure exemplifying the gating strategy is provided in the Supplementary Information.
